# Charting the Lipopeptidome of Nonpathogenic Pseudomonas

**DOI:** 10.1128/msystems.00988-22

**Published:** 2023-01-31

**Authors:** Catherine Cesa-Luna, Niels Geudens, Léa Girard, Vic De Roo, Hassan R. Maklad, José C. Martins, Monica Höfte, René De Mot

**Affiliations:** a Centre of Microbial and Plant Genetics, Faculty of Bioscience Engineering, KU Leuven, Heverlee-Leuven, Belgium; b NMR and Structure Analysis Unit, Department of Organic and Macromolecular Chemistry, Faculty of Science, Ghent University, Ghent, Belgium; c Laboratory of Phytopathology, Department of Plants and Crops, Faculty of Bioscience Engineering, Ghent University, Ghent, Belgium; UMR1136 INRA Université de Lorraine

**Keywords:** biosynthetic gene clusters (BGCs), cyclic lipopeptides (CLPs), genome mining, lipopeptidome, NMR fingerprint matching, nonribosomal peptide synthetases (NRPSs), phylogeny, *Pseudomonas*, rpoD

## Abstract

A major source of pseudomonad-specialized metabolites is the nonribosomal peptide synthetases (NRPSs) assembling siderophores and lipopeptides. Cyclic lipopeptides (CLPs) of the Mycin and Peptin families are frequently associated with, but not restricted to, phytopathogenic species. We conducted an *in silico* analysis of the NRPSs encoded by lipopeptide biosynthetic gene clusters in nonpathogenic Pseudomonas genomes, covering 13 chemically diversified families. This global assessment of lipopeptide production capacity revealed it to be confined to the Pseudomonas fluorescens lineage, with most strains synthesizing a single type of CLP. Whereas certain lipopeptide families are specific for a taxonomic subgroup, others are found in distant groups. NRPS activation domain-guided peptide predictions enabled reliable family assignments, including identification of novel members. Focusing on the two most abundant lipopeptide families (Viscosin and Amphisin), a portion of their uncharted diversity was mapped, including characterization of two novel Amphisin family members (nepenthesin and oakridgin). Using NMR fingerprint matching, known Viscosin-family lipopeptides were identified in 15 (type) species spread across different taxonomic groups. A bifurcate genomic organization predominates among Viscosin-family producers and typifies Xantholysin-, Entolysin-, and Poaeamide-family producers but most families feature a single NRPS gene cluster embedded between cognate regulator and transporter genes. The strong correlation observed between NRPS system phylogeny and *rpoD*-based taxonomic affiliation indicates that much of the structural diversity is linked to speciation, providing few indications of horizontal gene transfer. The grouping of most NRPS systems in four superfamilies based on activation domain homology suggests extensive module dynamics driven by domain deletions, duplications, and exchanges.

**IMPORTANCE** Pseudomonas species are prominent producers of lipopeptides that support proliferation in a multitude of environments and foster varied lifestyles. By genome mining of biosynthetic gene clusters (BGCs) with lipopeptide-specific organization, we mapped the global Pseudomonas lipopeptidome and linked its staggering diversity to taxonomy of the producers, belonging to different groups within the major Pseudomonas fluorescens lineage. Activation domain phylogeny of newly mined lipopeptide synthetases combined with previously characterized enzymes enabled assignment of predicted BGC products to specific lipopeptide families. In addition, novel peptide sequences were detected, showing the value of substrate specificity analysis for prioritization of BGCs for further characterization. NMR fingerprint matching proved an excellent tool to unequivocally identify multiple lipopeptides bioinformatically assigned to the Viscosin family, by far the most abundant one in Pseudomonas and with stereochemistry of all its current members elucidated. In-depth analysis of activation domains provided insight into mechanisms driving lipopeptide structural diversification.

## INTRODUCTION

The exploration of environmental microbiomes for useful bioactivities has revealed that certain bacterial phyla stand out by production of a plethora of specialized metabolites. Much of this chemical diversity is attributed to modular mega-enzymes capable of assembling small building blocks into complex molecules. Whereas biosynthetic principles of fatty acid (FA) synthesis are adopted in polyketide synthesis, the ribosomal formation of peptide bonds is mimicked by nonribosomal peptide synthetases (NRPSs). NRPS systems generate a significant part of the specialized metabolome in phyla, such as *Actinobacteria*, *Cyanobacteria*, and *Firmicutes* (1). Among the *Proteobacteria*, NRPS-proficient genera include *Burkholderia*, *Xenorhabdus*, and Pseudomonas (2–6). NRPS systems mediating synthesis of cyclic or linear lipopeptides (CLPs or LLPs) are frequently present in isolates hailing from soil and plant environments (7–10). The large majority of nonphytopathogenic lipopeptide (LP)-producing pseudomonads possess a single type of LP system. These “mono-producers” secrete one or more congeners of an LP belonging to a particular chemical family in which members can show limited variation in linear (un)saturated FA and/or in amino acid (AA) sequence/stereochemistry of a peptide with defined length (see Table S1 in the supplemental material). Only few nonphytopathogenic “dual producers” are known to cosynthesize CLPs belonging to two chemically distinct families (11, 12). Strains carrying clustered biosynthetic gene clusters (BGCs) of the Mycin and Peptin families, along with a third BGC for either an LLP of the Syringafactin family (13), the LLP thanafactin (14), or asplenin (15), are confined to particular taxonomic (sub)groups harboring both pathogenic and phytobeneficial isolates (Table S2) (16, 17). Biosynthesis of the cyclic lipononapeptides of the Mycin family requires the activity of a didomain enzyme for chlorination and incorporation of a terminal chlorothreonine (18). Such standalone enzymes (type II NRPS) are not involved in assembly of other Pseudomonas LPs, including the Peptins (CLPs composed of 19, 22, or 25 AAs). Their synthesis is mediated by type I NRPSs, large modular enzymes that harbor all the necessary enzyme activities. More specifically, Pseudomonas LPs can be classified in distinct families, based on their total AA sequence length and the size of the macrocycle that is formed between the C-terminal carboxyl and a hydroxyl moiety in the side chain of a preceding AA (8, 16).

10.1128/msystems.00988-22.8 TABLE S1 Family classification and chemical structures of LPs from nonpathogenic isolates. Per family, the characteristic combination of peptide length (l) and macrocycle size (m) is indicated as [l:m]. For peptides with resolved stereochemistry the amino acid (AA) configuration is specified. The AAs involved in the macrocycle, if present, are shaded. The position and extent of this ring structure is marked by a green bar. Family names are denoted with capital first letter (Viscosin, etc.) and individual member names without capital (viscosin, etc.). BGC accession numbers and references are indicated in Table S3. Download Table S1, PDF file, 0.1 MB. Copyright © 2023 Cesa-Luna et al. 2023 Cesa-Luna et al. https://creativecommons.org/licenses/by/4.0/ This content is distributed under the terms of the Creative Commons Attribution 4.0 International license.

10.1128/msystems.00988-22.9 TABLE S2 Taxonomic affiliation of coproducers of Mycin and Peptin LPs. Colored shading indicates by which Pseudomonas group (G) or subgroup (SG) specific LPs of the Mycin (blue) and Peptin (green) families are produced. A tentative structure has been reported for thanapeptin. Download Table S2, PDF file, 0.05 MB. Copyright © 2023 Cesa-Luna et al. 2023 Cesa-Luna et al. https://creativecommons.org/licenses/by/4.0/ This content is distributed under the terms of the Creative Commons Attribution 4.0 International license.

**FIG 1:**
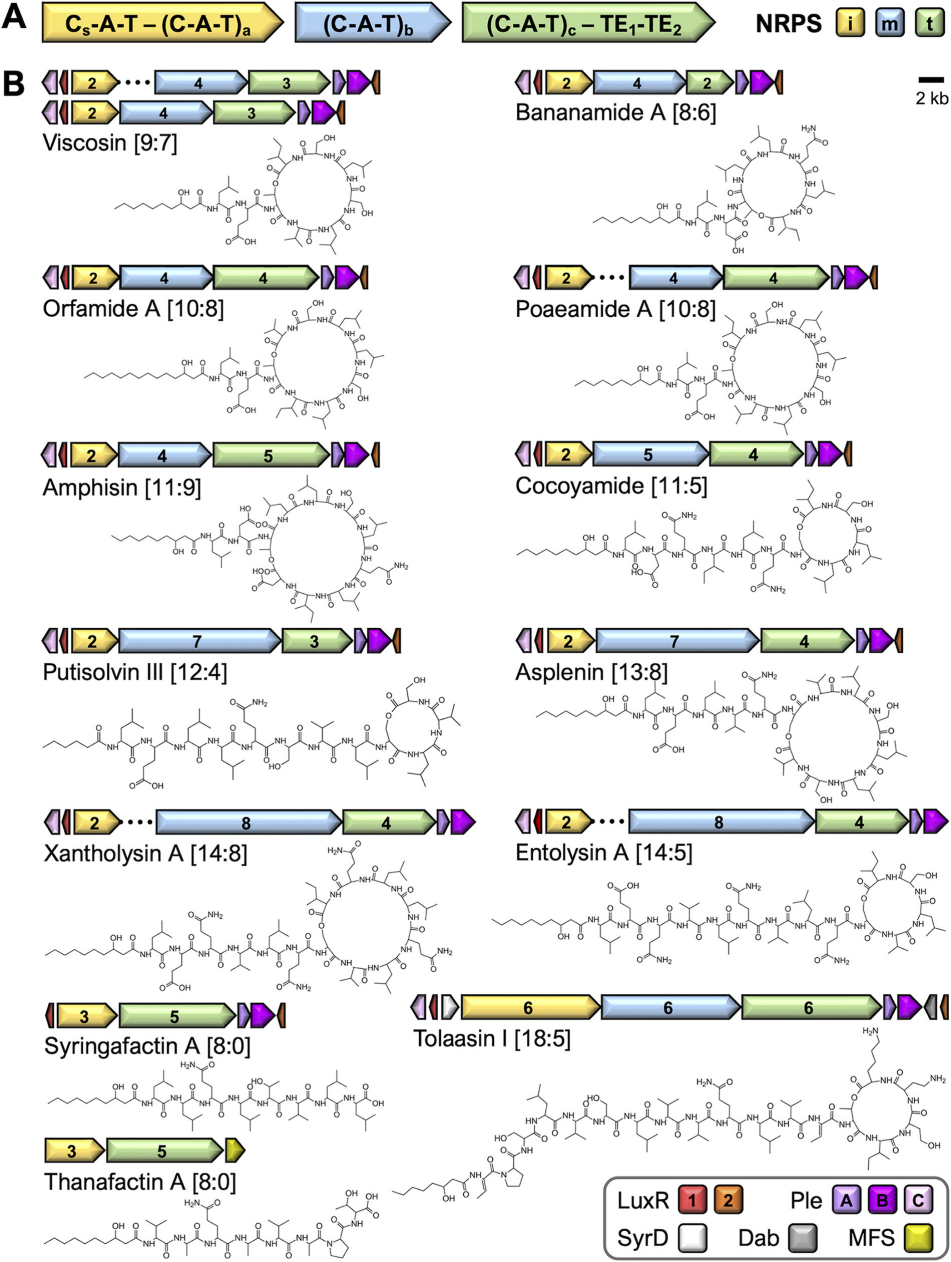
Structural and genomic diversity of LPs in nonpathogenic Pseudomonas. (A) Generic representation of the multidomain architecture of the NRPS enzymes synthesizing the majority of cyclic and linear LPs in Pseudomonas. Biosynthesis is initiated by NRPS^i^, continued by NRPS^m^ (if present), and completed by NRPS^t^. Assembly starts with acylation of the first AA by the C_s_-A-T module. Synthesis proceeds in a collinear fashion, matching the order of the consecutive C-A-T modules, by progressive extension of T domain-attached LP intermediates with A domain-selected residues. The linear or cyclic product (peptide length *n* = 1+a+b+c) is released by the thioesterase activity of the tandem TE domains. Unlike in the biosynthesis of Mycin-type CLPs, standalone NRPS or tailoring enzymes are not required. (B) Genomic architecture of the LP BGCs of different families (Table S1) with the NRPS genes flanked by one or two LuxR-family regulatory genes and three genes encoding the tripartite PleABC export system. An additional type of ATP-dependent transporter (SyrD) and an aminotransferase (Dab) for synthesis of 2,4-diaminobutyric acid substrate are encoded in the Tolaasin family BGCs. A conserved secondary transporter gene of the major facilitator superfamily (MFS) is associated with the thanafactin NRPS genes. The number of AA-specifying modules is indicated for each NRPS gene. In addition to the predominant contiguous BGC organization, a splitted cluster (represented by a dotted connector line) is typical for the Poaeamide, Entolysin, and Xantholysin families. Both types of organization occur in the Viscosin family.

BGC organization and NRPS architecture are well conserved across the “non-Mycin” LP families (8) (Fig. 1). An operon type of organization with all NRPS genes arranged in the order of the consecutive LP elongation activities they encode, is the most common (Fig. S1A). However, some families display a “splitted” BGC configuration, with the initiator NRPS gene at a genomic location distant from the operon with the other NRPS genes (Fig. S1B). Moreover, the genomic regions flanking the NRPS gene clusters, either contiguous or bifurcated, show a remarkable synteny of LuxR-family regulatory genes and transport genes (*pleABC*) encoding an ATP-dependent tripartite export system (19–21) (Fig. 1).

10.1128/msystems.00988-22.1 FIG S1 Organization of LP BGCs in nonphytopathogenic Pseudomonas. (A) Generic representation of the predominant organization with a single contiguous region comprising two or three genes encoding the consecutively acting NRPS enzymes N1, N2 (absent in linear LPs), and N3 (genes represented by arrows; not to scale). Biosynthesis is initiated by N1 and terminated by N3. The flanking regions (also not to scale) illustrate the widely conserved organization of transporter genes and regulatory genes. The black diamond symbol visualizes the location where seemingly split BGCs are separated and localized on different parts of the genome. The R1 and R2 products are cognate non-QS LuxR-family regulators. Proteins T1, T2, and T3 constitute the tripartite LP exporter system PleABC (T1, periplasmic adaptor protein PleA; T2, ATP-dependent transporter PleB; T3, outer membrane protein PleC). The number of C-A-T modules in the consecutive NRPSs and the resulting peptide length (number in square brackets) are specified for the respective families. Putative LPs predicted in this study are highlighted in blue font. Predicted LP4 (not shown) is derived from a single four-module NRPS. In dual producers of the Tolaasin family two unlinked trigenic BGCs are present (sessilin and orfamide) or a Tolaasin cluster is combined with a split cluster (shown in panel B) for pseudodesmin or viscosinamide (this study). Two additional genes are present in these Tolaasin BGCs: a *syrD* ABC transporter homolog (T4) and a gene encoding an aminotransferase (gene product D) for synthesis of 2,4-diaminobutyric acid (Dab). A notable exception for a split putisolvin BGC was predicted in the strain RW4S1. The distinct genomic island configuration of Peptin and Mycin BGCs is not represented here. (B) Representative genomic configurations of bifurcate organization with the N1 gene (orange) and the N2-N3 gene pair (blue-green) residing in different parts of the producer genome (genes drawn to scale). The upstream region of N1 accommodates the R1 and T1 genes and the downstream region of N3 encodes T1, T2 and − in most BGCs − R2 (same organization as in panel A). The genomic distance between both gene clusters (values in kbp; between square brackets) is specified for CLP systems of known producers (LP and/or BGC identified in this study with name in green font). If for those strains the inter-BGC distance is unknown, it is shown for a strain (name in dark blue) with a predicted orthologous NRPS system (corresponding distance marked with asterisk; actual producer strain name between rounded brackets). In the short 7-kbp region between the convergent COW39 xantholysin biosynthetic genes *xtlBC* and *xtlA*, its T1-T2 and R1-T3 genes have merged, being only separated by a regulator gene of the AraC family. In predicted viscosinamide producer *P. costantinii* strain 21815971, its putative N1 and N2-N3 genes are only 524 bp apart, approaching a trigenic BGC architecture (not shown). Download FIG S1, PDF file, 0.2 MB. Copyright © 2023 Cesa-Luna et al. 2023 Cesa-Luna et al. https://creativecommons.org/licenses/by/4.0/ This content is distributed under the terms of the Creative Commons Attribution 4.0 International license.

Biosynthesis of Pseudomonas type I NRPS products follows the collinearity rule, stating that the peptide sequence is determined by the order of the modules in the consecutively acting enzymes. The FA is attached to the first AA by a specialized condensation (C) domain (C_s_; starter). Subsequent AA additions are catalyzed by either a regular condensation domain (^L^C_L_) or, more frequently, by a condensation domain with built-in epimerization capacity (C/E). When functional, the latter mimics the activity of a ^D^C_L_ domain by converting the configuration of the carboxyterminal residue in the peptide intermediate from L to D before coupling it to the extender unit (22, 23). Separate epimerization domains are lacking in these NRPSs that are further characterized by a tandem of thioesterase domains (2, 8).

Due to the presence of dual-function C/E domains in their NRPS systems, Pseudomonas LPs are composed of both d- and l-amino acids, presenting a challenge for their structure elucidation. Moreover, the occurrence of epimerization-inactive C/E domains complicates this process further. In both respects, NMR fingerprint matching can provide a fast and unambiguous tool for the dereplication and/or elucidation of LP structures (https://www.rhizoclip.be; 24) and was applied here. Limiting the need for elaborate chemical analyses, NMR fingerprint matching only requires the comparison of the NMR spectrum of a newly isolated compound with that of available, previously described, reference compounds. When a match is obtained, the structural and, when available, stereochemical characterization of the LP is achieved. Its value is convincingly demonstrated in this work, as several newly isolated LP structures of Viscosin members could be confirmed without elaborate chemical analyses.

In this study, we exploit the conserved features of BGCs in nonpathogenic Pseudomonas to build an inventory of their collective lipopeptidome by NRPS system- and domain-level comparative analysis for producers of known LPs. Guided by A domain phylogeny, it is complemented with genomic prospection for genetically diversified candidate producers of known and novel LP family members, with ensuing chemical validation for selected representatives to extend the Pseudomonas lipopeptidome. In addition to predicting some novel LP families, this comprehensive study of genomic BGC organization and analysis of NRPS domain architectures combined with taxonomic assignment of producers provides insight in some of the evolutionary drivers of NRPS diversification within and between LP families.

## RESULTS AND DISCUSSION

### Pseudomonas lipopeptidome extended by genome exploration and mining.

**FIG 2:**
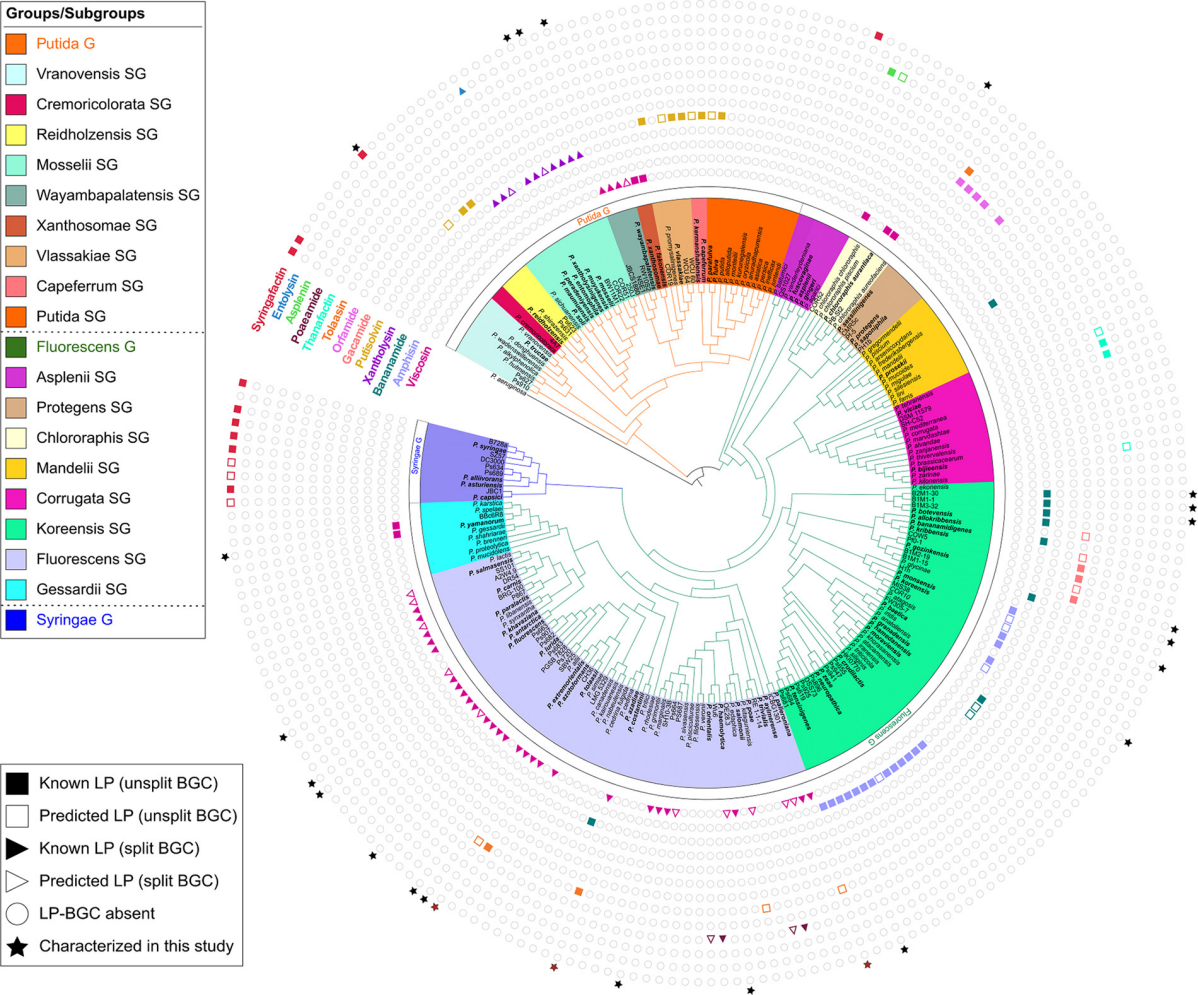
Global distribution of LP families in nonpathogenic Pseudomonas. The *rpoD* cladogram comprises previously characterized LP producers and related type strains, using P. aeruginosa as outgroup. This set is complemented with the LP producers identified in this study (marked with a black or red star to distinguish monoproducers and dual producers, respectively). Type strains highlighted with bold species names represent established and predicted LP producers. No (tentative) species names are included for nontype strains. All strains belong to either the Fluorescens, Putida, or Syringae group (green, orange, and purple branches in the cladogram, respectively). The subgroups constituting these lineages are delineated with different background colors as specified in the legend panel. The circumjacent annotation represents the capacity to produce an LP of a particular family, with families (colored names) differentiated by symbols of corresponding colors. Closed symbols refer to chemically identified LPs and open symbols designate predicted LP family members. Empty dots indicate that a LP BGC is absent (strains with published genome sequences) or that no evidence for LP production is available (strains without published genome sequences). The genomic BGC organization, contiguous or bifurcated, is specified by squares and triangles, respectively. The corresponding *rpoD*-based phylogenetic tree with bootstrap values is provided in Fig. S8 at https://doi.org/10.48804/KKFWS9.

**FIG 3:**
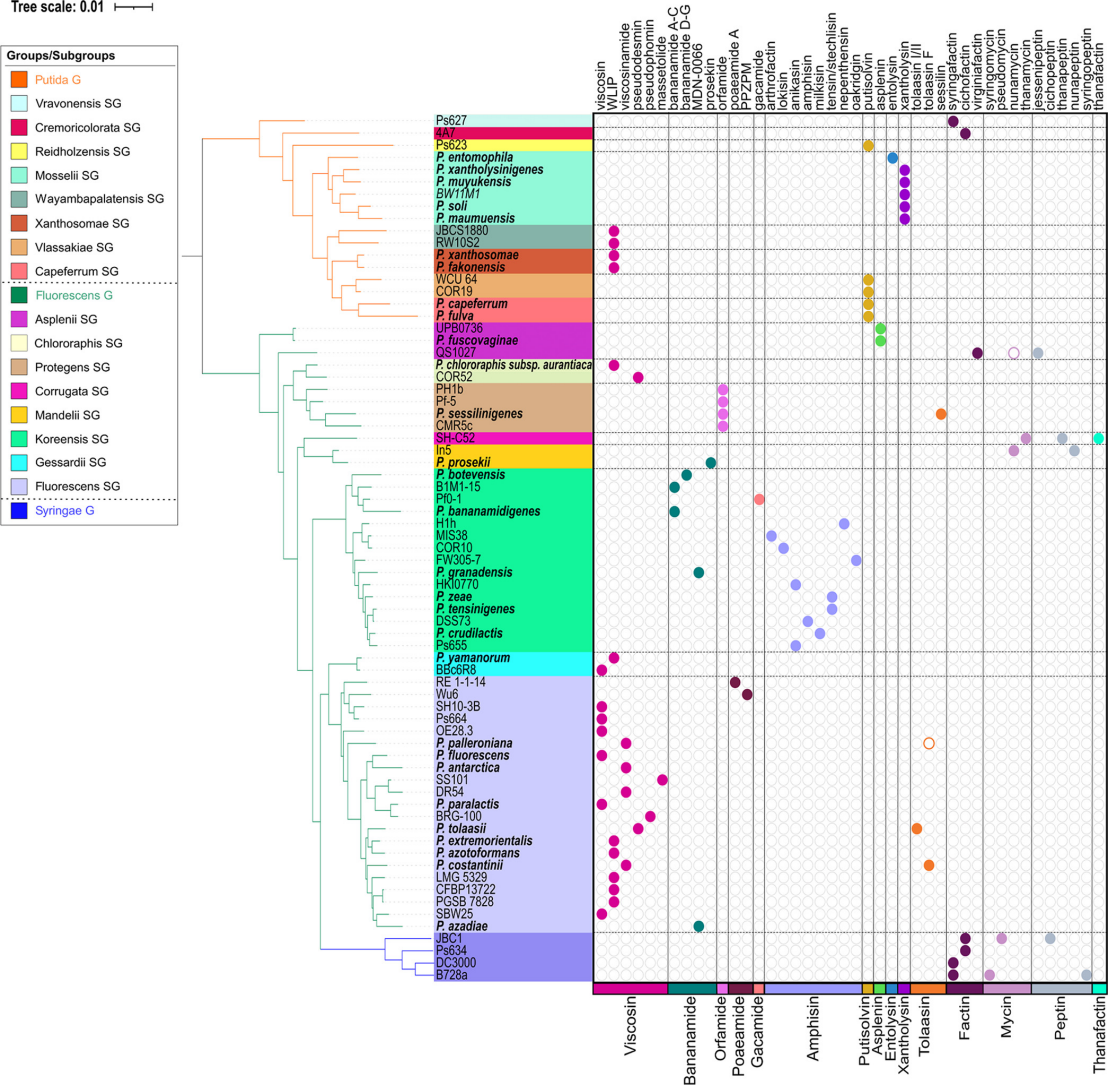
Affiliation of LP producers with Pseudomonas taxonomic (sub)groups. The demonstrated secretion of individual LPs is plotted by family-specific colored dots against an *rpoD*-derived phylogenetic tree of the corresponding producer strains, with the indication of the taxonomic (sub)group they belong to (bootstrapped tree in Fig. S9 at https://doi.org/10.48804/KKFWS9). All strains belong to either the Putida, Fluorescens, or Syringae group (orange, green, and purple tree branches, respectively). The subgroups constituting these lineages are delineated with different background colors as specified in the legend panel. Bold species names highlight LP-producing type strains. An empty colored dot indicates that an additional BGC of a specified family is present but that the predicted LP product awaits characterization. Selected producers of Mycin and Peptin CLPs are included for comparison.

**FIG 4:**
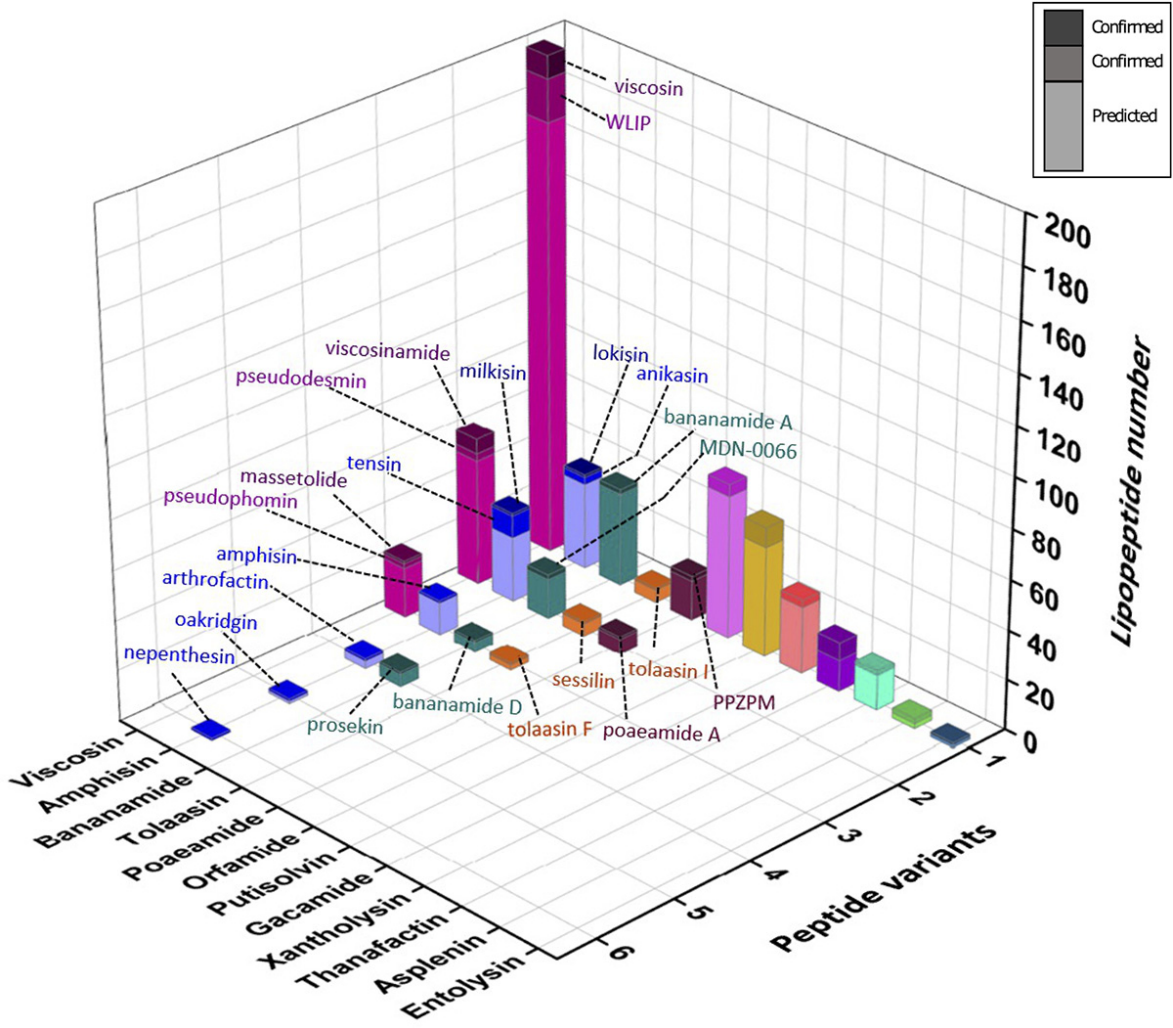
Quantitative distribution of LP BGCs in genomes of nonpathogenic Pseudomonas. The 3D bars represent numbers of known and predicted LP BGCs assigned to the respective known families. Within the families, LPs are further grouped along the *z* axis (1 through 6) according to peptide sequence if such variants occur. Stacked bars represent chemically characterized LPs (upper segment(s)) and predicted LPs (lower segment) as indicated in the legend box. In the Viscosin family, the upper bar segments correspond to the l-Leu5-containing LPs viscosin (peptide sequence variant 1), viscosinamide (variant 2), and massetolide (variant 3); the middle bar segments show the corresponding d-Leu5 stereoisomers WLIP (variant 1), pseudodesmin (variant 2), and pseudophomin (variant 3). Respectively, six and four sequence subtypes are shown for the Amphisin family (variants 1 to 6: lokisin/anikasin; milkisin/stechlisin/tensin; arthrofactin; amphisin; oakridgin; nepenthesin) and the Bananamide family (variants 1 to 4: bananamide A–C; bananamide D–G; MDN-0066; prosekin). Three sequence subtypes are shown for the Tolaasin family (variants 1 to 3: tolaasin I, sessilin, tolaasin F). The Poaeamide family comprises the sequence variants PPZPM (1) and poaeamide A (2). Minor congeners of a particular peptide sequence variant, if any, may be produced due to relaxed substrate specificity of a module, but these are not specified. The corresponding producer strains and accession numbers are compiled in Table S4.

LP-type BGCs retrieved from Pseudomonas genomes were sorted into families according to global NRPS module composition and peptide sequences predicted for the putative products based on phylogenetic clustering of extracted A domains in our data set of Pseudomonas A domains. For each family, the identified strains are compiled in Table S4A to N at https://doi.org/10.48804/KKFWS9. Potential producers of novel LPs are listed separately with the corresponding predicted peptide sequence (Table S4L). The presence of BGCs for 13 families is mapped on an *rpoD*-based taxonomic tree hallmarked with type strains (including LP-deficient species) from the Pseudomonas fluorescens lineage (Fig. 2 and Fig. S8 at https://doi.org/10.48804/KKFWS9). Profiling based on the *rpoD* gene, encoding the sigma 70 factor of RNA polymerase, is suitable for species-level differentiation in Pseudomonas, which cannot be achieved with 16S rRNA sequence analysis (25, 26). LP producers are absent from the Pseudomonas aeruginosa and Pseudomonas pertucinogena lineages. This tree is further populated with additional producers identified in this study, either of known or novel LPs. In most cases, stereochemistry could be assessed by NMR spectral matching (24). However, in cases where no reference NMR spectrum was available, the stereochemical make-up currently remains elusive. The taxonomic affiliation of individual producers (Fig. 3 and Fig. S9 at https://doi.org/10.48804/KKFWS9) shows a pronounced tendency of LP family-specific biosynthetic capacity being linked to one or few groups (Gs) or subgroups (SGs) of nonpathogenic Pseudomonas, clearly differentiated from those proficient in combined production of LPs of the Mycin and Peptin families that, in some species (e.g., P. syringae, *P. cichorii*), contribute to virulence (16, 17). The quantitative distribution of (potential) LP producers reveals large differences between families (Fig. 4). The Viscosin family, named after the first CLP discovered in Pseudomonas (27) represents by far the most abundant CLP type.

### Viscosin-related systems represent the most widespread LP BGCs in Pseudomonas.

**FIG 5:**
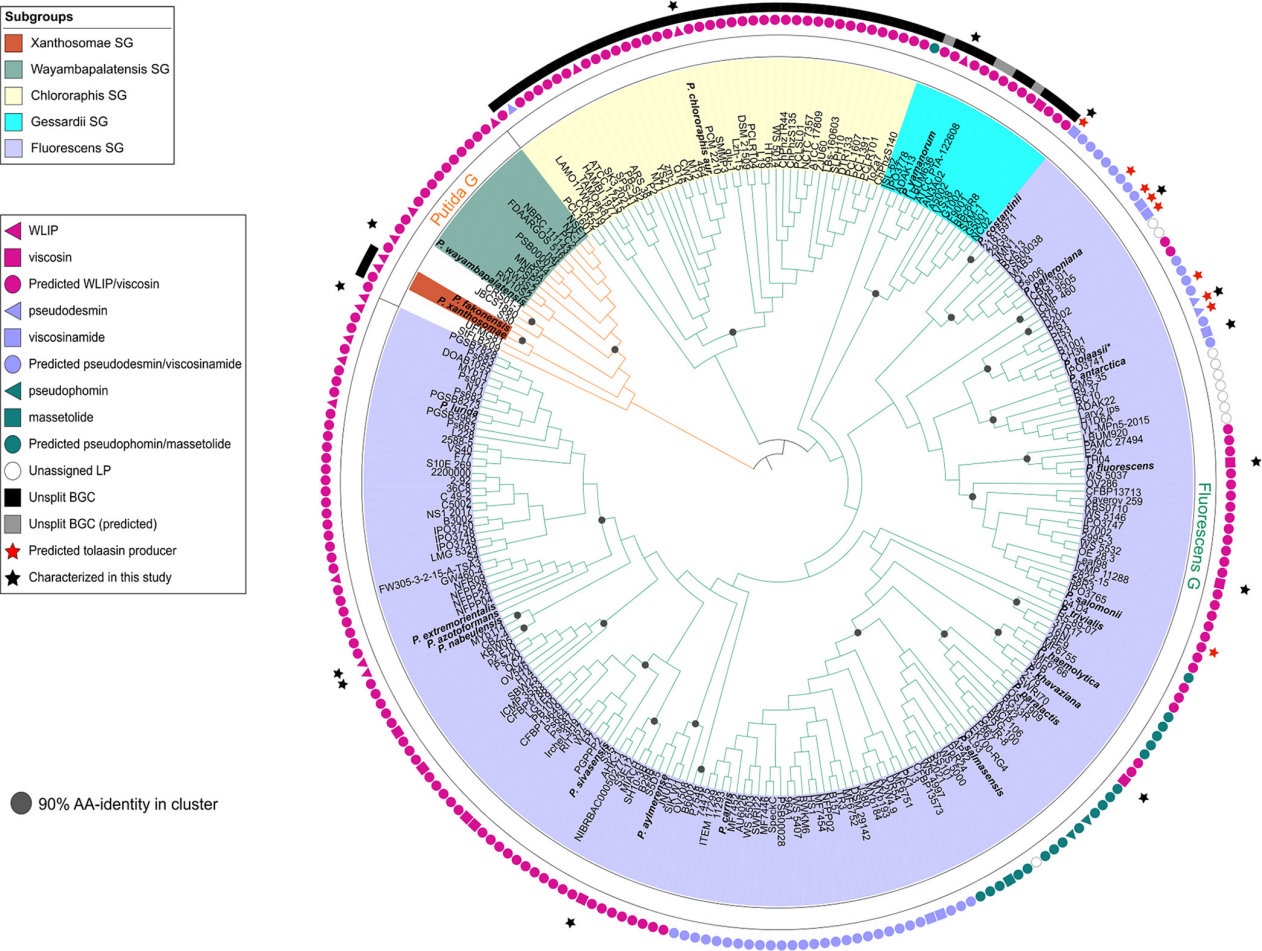
Diversity of NRPS systems in the Viscosin family. The NRPS-based cladogram comprises LP systems encoded by known and predicted Viscosin-family BGCs retrieved from (putative) LP producers. Type strains are highlighted with bold species names. Strain numbers are provided for all other isolates. The taxonomic (sub)group affiliations of the strains are specified with different background colors. The branches of the Putida G and Fluorescens G are highlighted in orange and green, respectively. Clusters of strains with NRPS concatenates that share at least 90% AA-identity are marked with a dark gray dot. The circular annotation of strains represents the capacity, either known or predicted, to produce a particular LP (specified in the legend panel). Different symbols are used for LPs containing l-Leu5 (squares) or d-Leu5 (triangles). The strains marked with a black strip carry a contiguous genomic BGC organization. A gray strip refers to such organization predicted by concatenation of two contigs, inferred from synteny with an orthologous system. The LP systems characterized in this study are marked with a black star. Strains that have the capacity to coproduce tolaasin (I or F) are highlighted with a red star. The corresponding tree inferred from an alignment of concatenated NRPS sequences, with bootstrap values added, is provided in Fig. S10 at https://doi.org/10.48804/KKFWS9.

We pursued CLP characterization by NMR matching for several taxonomically diversified strains with Viscosin-type NRPS sequences not closely clustering with previously characterized systems (Fig. 5; Fig. S10 at https://doi.org/10.48804/KKFWS9 and Table S4B). Thus, novel producers, among which several type strains, were identified for viscosin (3 strains), WLIP (7 strains), pseudodesmin (1 strain), and viscosinamide (4 strains, including Pseudomonas costantinii LMG 22119^T^). Since *P. costantinii* also synthesizes tolaasin F, a diastereoisomeric analog of tolaasin I (28), this reveals a novel CLP combination in a dual producer.

The Viscosin nonapeptidic CLPs comprise six distinct sequence types showing AA variation at only three positions (2/4/9) but being further differentiated by the stereochemistry of Leu5 (Table S1). Our peptide sequence predictions indicate that the Viscosin family is largely dominated by peptides with Val4, combined with either Glu2 (viscosin or WLIP) or Gln2 (viscosinamide or pseudodesmin). Only a minor proportion of strains have the capacity to produce the Ile4 peptides (massetolide or pseudophomin) (Fig. 4). Most producers are affiliated with the Fluorescens G (particularly its Fluorescens SG) but WLIP-producing Putida G strains occur also, suggesting convergent evolution (Fig. 3 and 5).

### The Koreensis subgroup houses the structurally diversified Amphisin family.

Coming second in estimated abundance are CLPs of the Amphisin family (Fig. 4). Its known members differ in three out of the 11 AAs (positions 8/9/11) (Table S1). The Amphisin family is dominated by LPs with a peptide sequence that is present in anikasin/lokisin or in tensin/milkisin (Table S4E). In addition to Pseudomonas *zeae* OE 48.2^T^ (19), Pseudomonas *tensinigenes* ZA 5.3^T^ was confirmed to biosynthesize tensin. A comparatively small number of potential arthrofactin producers and only a few candidate amphisin producers were detected. Four putative novel variants were identified in a few candidate monoproducers (Table S4E). These predictions were validated for two available strains: oakridgin is synthesized by Pseudomonas sp. FW305-7, isolated from groundwater (Oak Ridge, TN, USA; [29]); nepenthesin is produced by Pseudomonas sp. H1h, isolated from pitcher digestive fluid of the tropical carnivorous “monkey cup” plant, *Nepenthes* sp. (30). Nepenthesin stands out among other Gln-containing Amphisin members by the position switch of Gln6 and Ser8 (Table S1). All currently confirmed and predicted producers belong to the Koreensis SG (Fig. 3). The high level of congruence between the NRPS concatenate and *rpoD* phylogenies suggests that these LP systems tightly coevolved with divergence of their host species (see Fig. S11A to D at https://doi.org/10.48804/KKFWS9).

### Bananamide producers are affiliated with three different subgroups within the Fluorescens group.

Four known octapeptide variants in the Bananamide family arise by differences at three positions (2/6/8) (Table S1). This chemical diversification is reflected in the NRPS concatenate phylogeny (Fig. S11F), revealing distinct clusters for the CLPs produced by Pseudomonas *bananamidigenes* BW11P2^T^ (31), Pseudomonas *botevensis* COW3^T^ (32), and Pseudomonas granadensis LMG 27940^T^ (33), all affiliated with the Koreensis SG. MDN-0066 is also synthesized by a member of the Fluorescens SG, Pseudomonas *azadiae* SWRI103^T^ (24) (Fig. 3). The striking homology between the *P. azadiae* and *P. granadensis* MDN-0066 systems (Fig. S11F) suggests that the former was acquired via horizontal gene transfer (HGT) from a Koreensis SG donor. In multiple sugarcane rhizosphere isolates, we identified homologous BGCs along with putative BGC variants (LP7, LP10a, LP12) (Table S4L), suggesting the presence of a substantial Bananamide-proficient subpopulation being associated with this tropical crop (34). We recently identified prosekin of *P. prosekii* LMG 26867^T^ of the Mandelii SG as a fourth member synthesized by a quite divergent NRPS system (19). Next to the *P. prosekii* type strain and coisolated strains originating from the Antarctic region (35, 36), strain AALPS.10.MNAAK.13 isolated from the Arctic region carries a prosekin BGC (Table S4A) (37). However, this BGC is not confined to such cold-adapted strains as it is also present in bark beetle-associated isolate A2-NA12 (38). The arctic isolate N40(2020) carrying a divergent COW3-type BGC (Fig. S11F) bears witness of the diverse climatic environments occupied by Bananamide producers (39).

### Orfamide and Poaeamide families look similar chemically, yet quite different phylogenetically.

The NRPS systems assigned to the Orfamide family are confined to the Protegens SG (Fig. 3). The NRPS enzymes of Pseudomonas *sessilinigenes* CMR12a^T^ (11), Pseudomonas *aestus* CMR5c (40), and Pseudomonas sp. PH1b (24), an isolate from the same carnivorous plant niche as nepenthesin producer H1h (30), have diverged substantially from the prototypical P. protegens producers, apparently reflecting speciation (Fig. S11G). The smaller Poaeamide family features two subtypes with different bifurcate orientation (Fig. S1B) and specificity of the fourth NRPS module (Leu4 *versus* Ile/Val4), corresponding with distinct species affiliations (*P. poae* and P. orientalis, respectively; Table S4D) within the Fluorescens SG (Fig. 3; Fig. S11H). Based on their macrocyclization and very similar decapeptides (10:8), the Poaeamide CLPs might be considered Orfamide variants with a shorter FA tail (Table S1). However, the low NRPS similarity (<70% AA identity), combined with different acylation specificities and genomic organizations (bifurcate for Poaeamide versus unsplitted for Orfamide), suggests partially converging evolutionary tracks in distant taxonomic subgroups.

### Tolaasin producers frequently harbor a second distinct LP system.

Secretion of tolaasin I as a virulence factor is considered a hallmark of the mushroom pathogen Pseudomonas tolaasii (41). A tolaasin I analog with Gln6 substituting for Ser6, sessilin A, is synthesized by *P. sessilinigenes* CMR12a^T^ (11). Besides featuring highly similar structures, tolaasin I and sessilin A display antagonistic activity against oomycetes, influenced by alterations in membrane sterol composition (42). *P. costantinii* DSM 16734^T^ (LMG 22119^T^) secretes tolaasin F, differing from both tolaasin I and sessilin at two peptide positions: d-Val9 versus l-Val9 and l-Ile15 versus l-Leu15 (28). NRPS-based phylogeny reveals a dichotomy within the Tolaasin family (Fig. S5I) reflected in a different BGC organization (Fig. S1A). Both the CMR12a^T^ *sesABC* and NCPPB 2192^T^ *tolABC* operons encode a [6-6-6] modular NRPS configuration (11, 12), contrasting with the tolaasin F *taaABCDE* genes encoding a [3-3-3-3-6] configuration. The tolaasin F peptide sequence is also predicted for Pseudomonas palleroniana LMG 23076^T^ and Pseudomonas salomonii LMG 22120^T^ (Table S4J), both with a hybrid [3-3-6-6]-configured BGC (Fig. S1A). These module constitution variants suggest that the tripartite *tol* operon may have evolved through distinct gene fusion events. Based on the collinearity principle, a possible scenario for this would be: first, the fusion of the 3rd and 4th NRPS gene in the five-membered operon of *P. costantinii* giving rise to the *P. palleroniana*/*P. salomonii* BGC configuration; then, a further evolution of the latter operon by fusion of its 1st and 2nd NRPS gene yielding the *P. tolaasii*/*P. sessilinigenes* NRPS system.

10.1128/msystems.00988-22.5 FIG S5 Shared NRPS backbones in the Viscosin and Poaeamide families. Pairwise sequence alignment of concatenated NRPSs (yellow arrows) mediating biosynthesis of massetolide (Mass; SS101), PPZPM (Ppz; Wu6), or poaeamide A (Poa; RE*1-1-14). The C, A, T, and TE domains are delineated using colored rounded rectangles (aqua, light red, teal, and dark red, respectively). The C and A domains are numbered according to the position of the AAs incorporated in the respective LPs. AA similarity between aligned NRPS sequence pairs is differentiated by black (identical), grey (similar), white (not similar) representation (Blosum62 score matrix). The graph visualizes the pairwise sequence identity (sliding window = 50; 100%, green; 30 to 99%, green-brown, <30%, red). The region lacking in the shorter sequence of MassC (A) is highlighted in purple. The additional A domain in PpzC mediates incorporation of Leu8 in PPZPM. The structural diversification between poaeamide and PPZPM, attributed to the difference in specificity of the A4-domain in PoaB (Leu4) and PPZPM (Ile4), is apparent from the sequence identity drop coinciding with those A domains (B). The Greek symbols refer to proposed module addition (α) and module exchange (ε). Download FIG S5, PDF file, 0.2 MB. Copyright © 2023 Cesa-Luna et al. 2023 Cesa-Luna et al. https://creativecommons.org/licenses/by/4.0/ This content is distributed under the terms of the Creative Commons Attribution 4.0 International license.

Strikingly, the Tolaasin family comprises several dual CLP producers. A second BGC of the Viscosin family is present in members of the Fluorescens G: pseudodesmin in *P. tolaasii* (Hermenau et al. [12]; this study); viscosinamide in *P. costantinii* (Scherlach et al. [28]; this study) and *P. palleroniana* (this study); and predicted Viscosin-type CLP in *P. salomonii* (this study). Conversely, *P. sessilinigenes* CMR12a^T^ maintains a tolaasin-like BGC (or sessilin predecessor) to complement its orfamide BGC that is common to most of its fellow Protegens SG members. Both *ses* and *ofa* BGCs are found in *P. aestus* BW16M1. Protegens SG monoproducer soil isolates with a tolaasin BGC (strain H1F5C) or sessilin BGC (strain MSSRFD41) (Fig. S11I and Table S4J) (43), respectively, may have lost their original capacity to produce orfamide.

These mixed CLP profiles hint to the involvement of both gene gain and loss in shaping the specialized metabolomes of individual strains. The adoption of a member from a different chemical family may be facilitated by minimal overlap between the respective pools of building blocks. Indeed, one third of the AAs in Tolaasin CLPs are absent from Viscosin and Orfamide CLPs and proportionally 2-fold more valine substrate is required.

### Singletons contribute substantial LP diversity.

The Pseudomonas lipopeptidome diversity is further broadened by several individual CLPs not belonging to one of the previous families and without known module variants (Fig. 2). Gacamide (cocoyamide) is a (predicted) metabolite of Koreensis SG strains related to Pseudomonas (*allo*)*kribbensis* and Pseudomonas *gozinkensis* (44, 45) (Table S1, Table S4F, and Fig. S11M). Known and predicted producers of putisolvin (46), entolysin (47), or xantholysin (48) are affiliated with various subgroups of the Putida G (Table S1, Table S4.G to I, and Fig. S11N and O). Xantholysin-producing species displaying full NRPS-*rpoD* congruence include Pseudomonas mosselii (48), Pseudomonas soli (49), Pseudomonas *xantholysinigenes* (19), and Pseudomonas *muyukensis* (25) (Fig. S11O). A BGC in the *P. mosselii* Gil3 genome (50) lacks the equivalent of four entire modules (5 through 8) such that the inferred decapeptide (LP10b) would be devoid of a serine residue for cyclization (Table S4L).

Our A domain analysis for putisolvin (I, II) of P. putida PCL1445 (with Leu4, Leu8 [46]) is at odds with the reported structure (Ile4, Ile8) but in agreement with the putisolvin variants (III–V) described for Pseudomonas spp. COR19, *P. vlassakiae* WCU_60/WCU_64 (51) and *P. capeferrum* WCS358^T^ (this study). We recently identified Pseudomonas fulva as a novel putisolvin-producing species (19). Among others, additional candidate putisolvin producers include Pseudomonas parafulva, Pseudomonas reidholzensis, and Pseudomonas *kermanshahensis* type strains. For most (predicted) putisolvin producers, the NRPS phylogeny mirrors well the *rpoD* phylogeny (Fig. S11N) but congruence deviations are apparent for some strains, e.g., Ps631/Ps623 (52) and COR19/WCU 64 (51), which may be indicative of more recent exchanges of NRPS genes between related species within the Putida G.

### Thanafactin: an odd one out.

Limited chemical diversity is contributed to the Pseudomonas lipopeptidome by LLPs. Our genome exploration revealed no novel Syringafactin members but revealed a functional cichofactin BGC in strain 4A7 of the Putida G (Table S4N and Fig. S11P). Unlike most Syringafactin producers, but alike to P. syringae pv. *tomato* DC3000 (53) and some other P. syringae strains (17), it lacks Mycin and Peptin BGCs. Recent characterization of additional cichofactin and syringafactin monoproducers (Table S4N) suggests this LLP profile to be more widespread than previously thought (17, 52, 54). The thanafactin BGC bears no similarity of NRPSs and accessory genes to those of all other Pseudomonas LPs, including the Syringafactin family. Deviating from the predominant LP BGC organization, the thanafactin NRPS genes are clustered with only a single downstream gene encoding a pmf-driven major facilitator superfamily (MFS) transporter (14) (Fig. 1). Together with strong domain sequence divergence compared to all other LP NRPS systems in Pseudomonas (data not shown), this may reflect its original acquisition from an unrelated bacterium. It is only found in Corrugata SG strains (Fig. 3), albeit absent (possibly lost) from the *P. corrugata* type strain (Table S4N). In addition to most strains that carry both a thanafactin-like BGC and Mycin-Peptin island (16), a few putative thanafactin monoproducer genomes have also been sequenced, including the *P. bijieensis* type strain (55). In one of those strains, inactivation of the thanafactin BGC reduced biocontrol activity in a Botrytis cinerea-pepper pathosystem (56). Two independent isolates carry a distinct BGC encoding a single tetra-modular NRPS, equivalent to the first module of ThfA and the three last modules of ThfB and suitable for assembly of lipotetrapeptide LP4 (see Table S4L).

### Lipotridecapeptides represent an underexplored part of the Pseudomonas lipopeptidome.

A lipotridecapeptide BGC is present in the type strains of *P. fuscovaginae* and *P. asplenii* (Asplenii SG), known coproducers of Mycin and Peptin CLPs (syringotoxin and fuscopeptin, respectively, [16]) (Table S4K). This LP apparently corresponds to N5 (renamed asplenin) detected in other Asplenii-SG rhizosphere isolates (15, 51). We identified candidate producers of four other Asplenin members with a potential eight-membered macrocycle but different AA sequences (LP13a to d; Table S4L). *P. gingeri* LMG 5327^T^ (Asplenii SG), P. chlororaphis 14D6 (Chlororaphis SG), as well as Pseudomonas sp. BP8 and *P. tructae* JCM 33436^T^ (both Putida G) carry a BGC predicted to yield LP13a, LP13b, LP13c, and LP13d, respectively. Congruence analysis suggests that these NRPS systems evolved independently from each other (Fig. S11Q). Overall, the LP13 peptides align well with putisolvin’s dodecapeptide, seemingly extended with a hydrophobic residue (Ile or Val) (Table S1). The *P. gingeri* system displays a [1-8-4] modular architecture, deviating from the four others with a [2-7-4] BGC arrangement. Among the currently characterized LPs, another mono-modular initiating NRPS has not yet been described.

### BGC bifurcation is not strictly linked to the LP family or producer taxonomic affiliation.

An N-terminal acylated dipeptide intermediate, FA-Leu-Glx/Asp, assembled by a bimodular initiator NRPS, is conserved in nearly all the surveyed LP families, except for the Tolaasin and Syringafactin families and thanafactin (tri- or hexamodular) (Fig. 1; Table S1). In the Xantholysin, Entolysin, and Poaeamide families and most of the Viscosin family, the initiator NRPS gene is genomically separated from the remainder NRPSs genes (Fig. 1). This bifurcate organization is not strictly linked to specific taxonomic clades, as illustrated by the BGCs in producers of Entolysin/Xantholysin (Putida G) and Poaeamide (Fluorescens G). For WLIP, viscosin, and pseudodesmin both BGC architectures occur (Fig. 5). Among known and potential putisolvin producers, all belonging to the Putida G, a splitted BGC configuration such as found in *P. vlassakiae* RW4S1 (this study) appears to be very rare (Table S4G). The splitted genomic organization seems entirely absent from the other CLP families with the N-terminal Leu-Glx/Asp motif, produced by strains belonging to the Koreensis SG, Mandelii SG, or Protegens SG. In the trigenic clusters an intergenic untranslated region (between 150 and 300 bp) is maintained between the initiator and the middle NRPS genes, possibly serving a *cis*-regulatory function.

### Homology between A domains reveals NRPS similarities among LP families.

We zoomed further in on the A domains, being considered key drivers of NRPS evolution toward structural product diversification. The hypothesis that associated C domains coevolve with A domains has been challenged by recent studies involving different bacterial phyla (57, 58). We briefly examined this hypothesis by phylogenetic analysis of A and ^L^C_L_ domains extracted separately from C-A-T modules of characterized Pseudomonas LP NRPSs. Within this subset of selected A domains, those specific for a certain substrate tend to form well-separated clades encompassing the different LP families and taxonomic groups (Fig. S2A). However, the companion ^L^C_L_ domains display a dispersed distribution, essentially clustering according to chemical family and producer taxonomic affiliation (Fig. S2B). This result is in line with the poor congruence observed between A and C domain phylogenies in Pseudomonas pyoverdine NRPSs (57). So, with the objective to dissect the substrate-specifying role of the LP synthetases, further analyses were focused on A domains rather than C-A pairs.

10.1128/msystems.00988-22.2 FIG S2 Comparison of phylogenetic tree topologies for A and ^L^C_L_ domains. Phylogenetic tree of A and ^L^C_L_ from C-A-T modules. The rationale to not include C/E domains pertains to their hybrid structure enabling catalytic activity linked to two adjacent modules. A *n*th C/E domain catalyzes epimerization of peptidic AA_n-1_ (selected by the *n*-*1*th A domain) prior to its coupling to AA*_n_* (selected by the *n*^th^ A domain). Such coadaptation to a preceding A domain likely affects the C/E-to-A comparison within a module. (A) In the A domain tree, clusters are colored according to AA specificity. (B) The same colors are used for the associated ^L^C_L_ domains. The numbers (A1, …; C1, …) refer to the position of the respective module in the NRPS systems. The NRPS systems for different LPs are specified by the names of the gene products (see Table S4 at https://doi.org/10.48804/KKFWS9): Ams, amphisin; Ani, anikasin; Arf, arthrofactin; Ban, bananamide; Cif, cichofactin; Gam, gacamide; Etl, entolysin; Lok, lokisin; Mass, massetolide; Mdn, MDN-0066; Mlk, milkisin; Nep, nepenthesin; Oak, oakridgin; Ofa, orfamide; Pdm/Pse, pseudodesmin; Pmn, pseudophomin; Poa, poaeamide; Ppz, PPZPM; Pek, prosekin; Pso, putisolvin; Ses, sessilin; Ste, stechlisin; Syf, syringafactin; Taa, tolaasin F; Ten, tensin; Thf, thanafactin; Tol, tolaasin; Vif, virginiafactin; Vis/Visc, viscosin; Vsa/Vsm, viscosinamide; Wip/Wlc/Wlp/Wlc/Wlf/Wly/Wpp, WLIP; Xtl, xantholysin. Download FIG S2, PDF file, 1.7 MB. Copyright © 2023 Cesa-Luna et al. 2023 Cesa-Luna et al. https://creativecommons.org/licenses/by/4.0/ This content is distributed under the terms of the Creative Commons Attribution 4.0 International license.

A global analysis of A domain sequences retrieved from the synthetases of structurally elucidated LPs reveals homogeneous clusters entirely specific for leucine, valine, or serine but mixed clusters are apparent for some other AAs (Fig. S3). Subsets of sequences of A domains linked to a particular AA were then separately analyzed to infer AA-specific phylograms (see Fig. S12A to F at https://doi.org/10.48804/KKFWS9). A strictly Val-specific A domain set can be defined (Fig. S12A) but Val, and less frequently Leu, can also be recruited by a promiscuous A domain type that is primarily used to activate Ile (Fig. S12C). We observed such relaxed substrate specificity for oakridgin, in line with ample reports for other LPs (pseudodesmin, massetolide, putisolvin, xantholysin, bananamide, poaeamide, orfamide, stechlisin, syringafactin, and virginiafactin; Table S1). This phenomenon may even be more widespread when minor congeners went uncharacterized. The Gln-specifying domains are intertwined with those for Glu and Asp, yielding a complex tree topology (Fig. S12E) as observed previously for a smaller data set (31). Particularly, mixed clusters with Glu- and Gln-specifying A2 domains hamper some peptide sequence predictions involving distinction between Viscosin-type CLPs with Gln2 (viscosinamide, pseudodesmin) and other Viscosin members with Glu2. Similarly, A domains for either Glu or Asp cocluster in the Amphisin and Bananamide families and recruitment of both Asp and Glu has been shown for arthrofactin (A11) and stechlisin (A2) (Fig. S12E).

10.1128/msystems.00988-22.3 FIG S3 Global analysis of A domains in Pseudomonas LP NRPS systems. Tree derived from multiple sequence alignment of A domains extracted from NRPSs mediating biosynthesis of structurally characterized Pseudomonas LPs. The clades are colored according to the corresponding incorporated AA. Homogeneous clusters specific for individual AAs are apparent for leucine, valine, and serine. However, a mixed clade is obtained for threonine (including 2,3-dehydroaminobutyric acid, Dhb) and for glutamate-glutamine-aspartate. For several of the A domains in the isoleucine clade, relaxed substrate activity has been observed, with the additional incorporation of valine or leucine. The minor branches for Dab, homoserine, lysine, and proline and a divergent glutamine branch correspond to A domains from the Tolaasin family NRPSs. The topology of the major clades is detailed in Fig. S12 at https://doi.org/10.48804/KKFWS9. Download FIG S3, PDF file, 0.3 MB. Copyright © 2023 Cesa-Luna et al. 2023 Cesa-Luna et al. https://creativecommons.org/licenses/by/4.0/ This content is distributed under the terms of the Creative Commons Attribution 4.0 International license.

**FIG 6:**
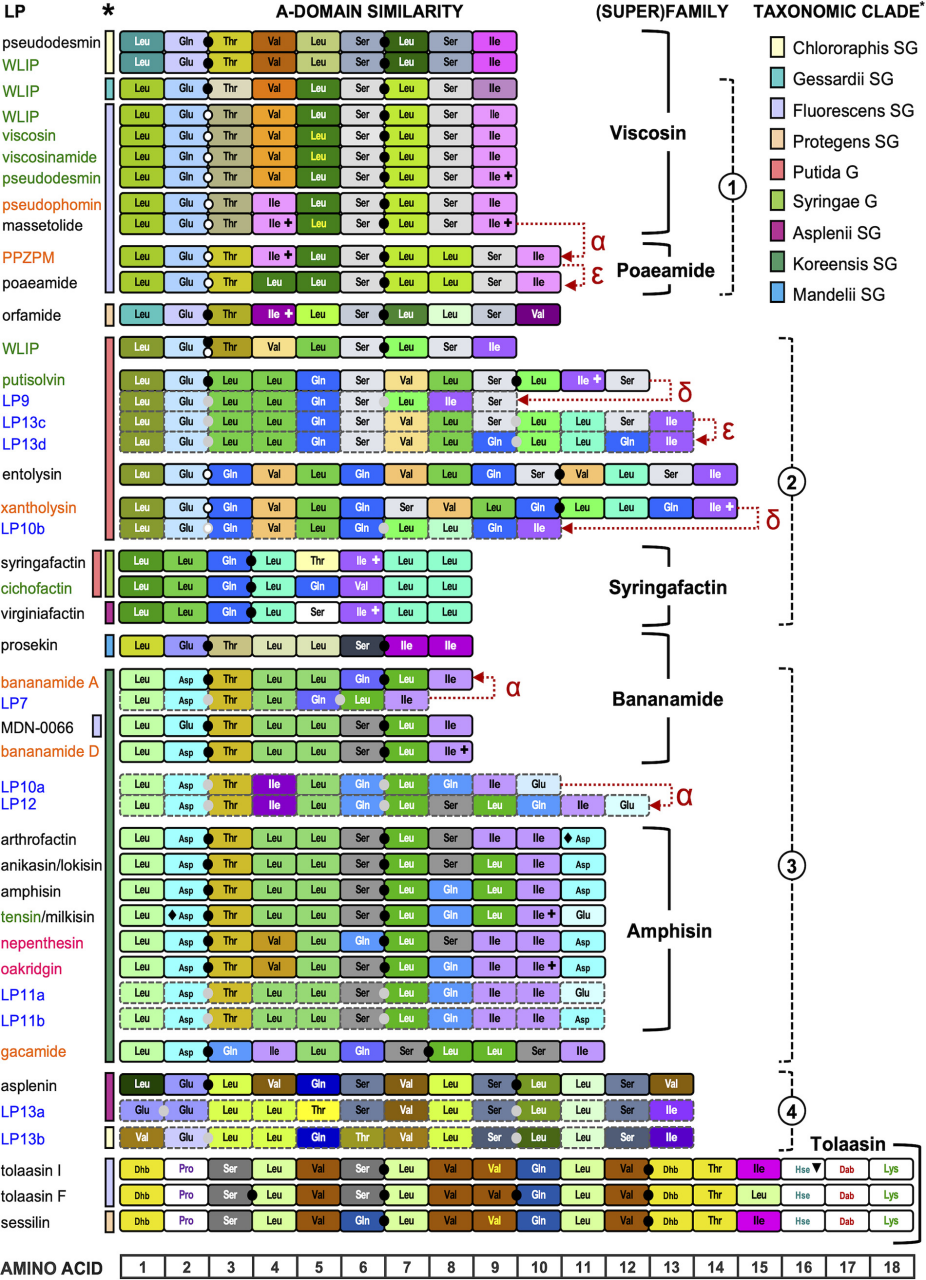
Relationships within and between LP families of nonpathogenic Pseudomonas revealed by A domain homology. Each A domain is represented by a box specifying its position in the NRPS (between 1 and 18) and selected AA. The resulting AA sequence(s) represent(s) the peptide sequence(s) found in the respective LPs (names shown on the left), assembled by collinear activity of the corresponding C-A-T modules in two or three consecutively acting NRPS enzymes (Fig. 1). A domains from modules present in different NRPSs constituting an LP system are separated by dots. The corresponding NRPS genes can be part of a contiguous BGC (marked with black/gray filled dots) or the first NRPS gene can occur separately (bifurcated BGC with split marked by an additional black/gray open dot). For systems with predicted LPs, dash-lined boxes and gray dots are used. To distinguish among domains that activate the same AA but belong to different homology clusters, box-filling colors are differentiated as defined in the respective phylogenetic trees for substrate AAs (Fig. S12). Boxes of A domains with relaxed substrate specificity are additionally labeled (plus sign, Ile-Val-Leu; diamond, Asp-Glu; triangle, Hse-Gly). AAs that occur only in the Tolaasin family are shown in a different color on a white background, except for Dhb (phylogenetically poorly separable from Thr-specific domains). Chemical families comprising multiple LPs are delineated with solid right brackets. Dashed right brackets with encircled numbers delineate four major superfamilies encompassing known and predicted LP systems sharing largely overlapping A domain homology profiles. LPs for which in this study the BGC of a previously reported producer strain is identified or the BGC of a novel producer strain/species is described, are shown with names in orange and green, respectively. LPs first described in this study are highlighted in pink (prediction validated by structure elucidation) or blue (prediction awaiting validation). A single A domain profile is used to represent a particular LP that is synthesized by orthologous NRPS systems in related species. Conversely, to indicate the involvement of sequence-diversified NRPS systems in biosynthesis of a particular LP by taxonomically distant producers, these are shown separately (pseudodesmin, WLIP). In the Amphisin family, peptide sequences that only differ by stereochemistry are combined (anikasin/lokisin; tensin[stechlisin]/milkisin). The stereochemical differentiation among members of the Viscosin family (by Leu5) and between Tolaasin CLPs (by Val9) is highlighted by using yellow font for the l-configured AAs. Colored vertical bars delineate the taxonomic (sub)group affiliation of the LP producers (specified in the taxonomic clade legend). Putative events evolutionarily linking certain known or predicted LPs (discussed in the text) are marked with red symbol-labeled dashed arrows representing module/domain addition (α), exchange (ε) or deletion (δ).

Considerable sequence divergence is apparent within AA-specific clades (Fig. S12A to F). Domain sequences sharing only moderate homology can still specify the same substrate, e.g., AA-identity of about 65% (Ser, Thr) or 60% (Leu, Ile, Val) and down to about 50% in particular modules (e.g., Gln A domains in the Tolaasin family versus those of the other families). To visualize A domain sequence divergence within these clades, different colors were assigned to distinct subclusters (Fig. S12A to F). This color scheme was then transposed to the corresponding modules to map similarity of NRPS systems within and across chemical families against a background of taxonomic affiliation of the corresponding producers (Fig. 6). The resulting patterns allowed to group LP systems sharing related A domains and we further ordered them by alignment of the synthesized peptide sequence. The patterns reflecting A domain diversification shown in Fig. 6 revealed underlying similarities between the NRPSs used by chemically different families.

### Chemically distinct LP families can be derived from related NRPS systems across taxonomic borders.

Families that one would not readily associate by mere comparison of peptide lengths/sequences and macrocycle sizes but exhibiting pronounced NRPS module homology can be considered part of a genetic/biochemical superfamily (or clan) based on their shared use of closely related A domains (Fig. 6). The Poaeamide systems (10:8) and those of the Viscosin family (9:7) encoded by Fluorescens SG and Gessardii SG strains can be assigned to superfamily 1. They are clearly differentiated from the Viscosin systems present in Chlororaphis SG (WLIP, pseudodesmin) or Putida G producers (WLIP), whose A domains seem to have coevolved with their respective hosts. The WLIP synthetases in the Putida G show prominent A domain homology with the NRPSs of putisolvin (12:4), entolysin (14:5), and xantholysin (14:8), predicted LP9, LP13c and LP13d, and the Syringafactin family, collectively defining superfamily 2, that accommodates multiple Putida G producers and some strains from the Syringae G and Asplenii SG. Superfamily 3 consists of the Bananamide (8:6) and Amphisin (11:9) members together with the predicted LP7, LP10a, and LP12 systems. In addition, several similar A domains of superfamily 3 are also shared by the gacamide (11:5) NRPSs. The entire population of superfamily 3 producers consists of *P. koreensis* SG strains, excluding the Bananamide member prosekin produced by a Mandelii SG species. Superfamily 4 can be proposed to unite predicted lipotridecapeptides from the Asplenii SG (asplenin and LP13a) and Chlororaphis SG (LP13b). In addition to the prosekin A domains, those of WLIP/pseudodesmin (in Chlororaphis SG), orfamide (Protegens SG), and tolaasin/sessilin (Fluorescens and Protegens SGs) have diverged substantially from each other and from the A domains in the four proposed superfamilies.

### NRPS systems hosted by unrelated strains can synthesize chemically identical LPs.

Previously, production of WLIP was already described for strains belonging to three well-separated taxonomic clades: (i) Putida G (44, 59, 60), (ii) Fluorescens SG (61), and (iii) Chlororaphis SG (62). In this study, we identified seven additional WLIP producers assigned to one of these clades (Fig. 3). In addition, we identified Gessardii as a fourth (sub)group, harboring WLIP-proficient Pseudomonas yamanorum LMG 27247^T^, a strain related to viscosin producer BBc6R8 (63) (Fig. 5). Production of pseudodesmin was reported for P. chlororaphis COR52 (64) and, as a second CLP, for tolaasin producer *P. tolaasii* NCPPB 2192^T^ (12). We also identified pseudodesmin in *P. tolaasii* CH36.

At first glance, the prominent similarity of the A domains in the Syringafactin NRPSs with those of putisolvin, xantholysin, entolysin, and all CLPs of P. putida G producers (Fig. 6; Fig. S4), seems at odds with the taxonomic position of the prototypical producers of syringafactin and cichofactin, respectively, P. syringae and *P. cichorii* (Syringae G) and of virginiafactin (Asplenii G). However, the isolation of syringafactin producers from a potato field (52) and our characterization in this study of a cichofactin producer originating from a built environment (strain 4A7, isolated from dust in an indoor gym facility [65]), along with our identification of a putative cichofactin BGC in *P. monteilii* strains (Table S4M and Fig. S11P) — all belonging to the Putida G (Fig. 3) — suggest that members of the Syringae G and Asplenii G originally acquired Factin-like BGCs from a Putida G donor (Fig. S4). This hypothesis would also explain cichofactin production by *P. asturiensis* and P. viridiflava strains (17, 52) and predicted cichofactin biosynthetic capacity for several related species within the Syringae G (Table S4M and Fig. S11P).

10.1128/msystems.00988-22.4 FIG S4 Relatedness between A domains in Syringafactin LPs and CLPs from producers of the Putida group. The cladogram is derived from multiple sequence alignment of A domains specifying Leu, Gln or Ile-Val, common to characterized cichofactin (Cif), syringafactin (Syf), and virginiafactin (Vif) producer strains (highlighted in red), along with the most similar corresponding A domains as identified in the AA-specific phylograms of Fig. S12. Those neighboring clusters comprise A domains predominantly found in producers of the Putida G (putisolvin [Pso, green], WLIP [purple], entolysin [pink], and xantholysin [blue]). Somewhat more distant clusters included (shown in black) originate from the Fluorescens G, namely Koreensis SG (bananamides, Ban; MDN-0066, Mdn; gacamide/cocoyamide, Gac/Coc), Mandelii SG (prosekin, Pek), and from the Asplenii G (gingeramide, Gin). The counterparts of Gln5 in cichofactin are Thr5 in syringafactin and Ser5 in virginiafactin. The SyfB-Thr5 A domains (B728a and DC3000) do not show similarity to a particular A-domain of the Pseudomonas CLP families included in Fig. S11. The VifB-Ser5 A-domain (QS1027) shares significant homology with the Ser-specifying domains of jessenipeptin (not included in Fig. S12). Download FIG S4, PDF file, 2.0 MB. Copyright © 2023 Cesa-Luna et al. 2023 Cesa-Luna et al. https://creativecommons.org/licenses/by/4.0/ This content is distributed under the terms of the Creative Commons Attribution 4.0 International license.

10.1128/msystems.00988-22.6 FIG S6 Predicted LPs sharing NRPS backbones with the Bananamide family. Pairwise sequence alignment of concatenated NRPSs (yellow arrows) mediating biosynthesis of bananamide (BW11P2) and predicted LP7 (B27[2017]), LP10 (R5[2017]), and LP12 (B2[2017]). The C, A, T, and TE domains are delineated using colored rounded rectangles (aqua, light red, teal, and dark red, respectively). The C and A domains are numbered according to the position of the AAs incorporated in the respective LPs. AA similarity between aligned NRPS sequence pairs is differentiated by black (identical), grey (similar), white (not similar) representation (Blosum62 score matrix). The graph visualizes the pairwise sequence identity (sliding window = 50; 100%, green; 30 to 99%, green-brown, <30%, red). The regions lacking in the shorter sequence are highlighted in purple. The additional A domains specify incorporation of Leu5 by BW11P2 BanB in bananamide (A), Gln8 and Glu10 (by prediction) in LP10 (B), and Ser8 and Leu9 in LP12 (by prediction) (C). The structural diversification between BW11P2 bananamide and LP10/LP12 by altered specificity of the A4 domain in the latter NRPS-2 enzymes (Leu *versus* Ile, respectively), is apparent from the sequence identity drop coinciding with those A-domains (C). The α symbol refers to module addition. Download FIG S6, PDF file, 0.3 MB. Copyright © 2023 Cesa-Luna et al. 2023 Cesa-Luna et al. https://creativecommons.org/licenses/by/4.0/ This content is distributed under the terms of the Creative Commons Attribution 4.0 International license.

### Comparative analyses of related NRPS systems reveal possible mechanisms of diversification.

Most LPs of the nonphytopathogenic Pseudomonas collective are built from a limited number of proteinogenic AA substrates, namely, the hydrophobic branched AAs leucine, valine, and isoleucine, the hydroxy-AAs serine and threonine, the acidic AAs glutamate and aspartate, and the basic AA glutamine. Despite this limited set of building blocks and without implementation of tailoring activities, a considerable variety of LPs is assembled by different NRPS systems. This enzymatic diversity bears witness of extensive domain dynamics. Conceivably, domain duplication within a producer strain can contribute to LP diversification. The presence of adjacent near-identical A domains encoded by a NRPS gene hints to such an event and presumptive duplications are found in most LP families (Fig. 6). Support for such a mechanism is provided when a candidate predecessor/successor BGC combination (known or predicted) can be identified. For instance, the PPZPM branch in the Poaeamide family may have evolved from the Viscosin-type CLPs massetolide or pseudophomin by extension with one residue (Leu8) through a Leu7 module duplication within the third NRPS gene (Fig. S5A). Likewise, a Leu4-module duplication in the second LP7 NRPS may explain the presence of the Leu4-Leu5 dipeptide in bananamide of strain BW11P2 (Fig. S6A). Further diversification within the Poaeamide family can be envisaged as the result of exchanging the 4th module in the second NRPS gene with a duplicate of the 5th Leu-specific module (Fig. 6; Fig. S5B). In this scenario poaeamide A would have evolved from PPZPM.

Indications for the diversification by deletion of multiple modules can also be found, although functionality of the affected BGCs requires characterization of the predicted products (LP4, LP9, LP10b). LP9 can be considered the result of a neat excision of three modules from the putisolvin gene *psoB* (Fig. 6; Fig. S7A) and deletion of four modules from a *P. mosselii* xantholysin gene *xtlB* would potentially produce the LP10b BGC (Fig. 6; Fig. S7B). The formation of the LP4 BGC can be attributed to recombination between *thfA* (Val1) and *thfB* (Val5) and ensuing excision of four internal AAs in thanafactin (Table S4N). A similar scenario was proposed for the evolution of cyanobacterial nodularins through deletion of two modules in a microcystin synthetase gene (66).

10.1128/msystems.00988-22.7 FIG S7 Predicted LPs with NRPS backbones reduced by module deletion. Pairwise sequence alignment of concatenated NRPSs (yellow arrows) mediating biosynthesis of: (A) putisolvin (COR19), predicted putisolvin (CC120222-01b) and predicted LP9 (CC120222-01a); (B) xantholysin (BW11M1) and predicted LP10b (Gil3). The C, A, T, and TE domains are delineated using colored rounded rectangles (aqua, light red, teal, and dark red, respectively). The C and A domains are numbered according to the position of the AAs incorporated in the respective LPs. AA similarity between aligned NRPS sequence pairs is differentiated by black (identical), grey (similar), white (not similar) representation (Blosum62 score matrix). The graph visualizes the pairwise sequence identity (sliding window = 50; 100%, green; 30 to 99%, green-brown, <30%, red). The regions lacking in the shorter sequence are highlighted in purple. The apparent deletions (δ) can be rationalized by recombinational excision involving homologous module (M) sequences: (A) M6(Ser) and M9(Ser) both encoded by *psoB*; (B) M5(Leu) and M9(Leu) both encoded by *xtlB*. Upon finalization of this analysis the LP10b BGC was identified in an independent isolate, BML-PP044 (GenBank accession numbers: BQIL01000025 and BQIL01000006). Download FIG S7, PDF file, 0.5 MB. Copyright © 2023 Cesa-Luna et al. 2023 Cesa-Luna et al. https://creativecommons.org/licenses/by/4.0/ This content is distributed under the terms of the Creative Commons Attribution 4.0 International license.

Baunach et al. (58) identified recombination as a major mechanism for NRPS diversification in cyanobacteria, involving module sequences from the same species but also from different species. Evidence supporting the hypothesis that the Ser5 module in virginiafactin of Pseudomonas sp. QS1027 was recruited from its jessenipeptin BGC has been reported (13). Among Pseudomonas BGCs (including Peptins and Mycins CLPs), we could not identify a possible source of the Thr5-specifying A domain in syringafactin (data not shown). We found no indications for a (recent) module acquisition from a cohosted BGC in any of the nonphytopathogenic (known and predicted) dual CLP producers, suggesting that this is not a common mechanism in Pseudomonas.

Clustering of NRPS modules among producers of chemically distinct CLPs within a (super)family suggests shared or overlapping ancestry and enables the proposition of evolutionary events that may have shaped intrafamilial chemical diversification or even generated a novel but chemically still similar family. Some examples are outlined below. Most A domains for the biosynthesis of gacamide are homologous to those used in the Amphisin family. Although of the same length, the gacamide peptide sequence is extensively reshuffled and imposes a pentacyclic structure instead of the nonapeptide ring of the Amphisin members. Peptide sequence similarity of the last nine AAs and same cyclization pattern rather relate gacamide structurally to entolysin. Based on the prominent similarity of the Bananamide NRPS domains with those of multiple sugarcane isolates predicted to assemble LP7, LP10a, or LP12, the latter LP set can also be provisionally assigned to the Bananamide-Amphisin-Gacamide superfamily 3 (Fig. 6). Possible events linking those LPs with the Gln-containing bananamide of strain BW11P2 include, in addition to a Leu4-domain duplication in LP7 (Fig. S6A), consecutive acquisitions of two extra modules (Gln8-Glu10 in LP10a and subsequently Ser8-Leu9 in LP12) (Fig. S6B and C). Bananamide diversification features a Gln-for-Ser (or reverse) substitution. Such a “Q-S switch” is also present in LPs of the Factin (virginiafactin versus cichofactin), Tolaasin (sessilin versus tolaasin), and Amphisin families (involving AA6 and AA8). It also differentiates entolysin from xantholysin at two positions (AA10 and AA13) and LP13c from LP13d (AA9 and AA12, by prediction) (Fig. 6). Compared to the frequent occurrence of the hydrophobic residues valine, leucine, or isoleucine at certain positions, a Ser-Gln substitution is not obvious to rationalize based on chemical structure and properties. However, the Ser-specifying A domain clade is the closest phylogenetical neighbor of those selective for Gln and such sequence similarity may facilitate recombinational exchange. Remarkably, in nepenthesin the Gln-Ser modules occupy positions opposite to those of all other known and predicted Gln-containing Amphisin members (Fig. 6). On the other hand, oakridgin and nepenthesin share a similar Val4 domain instead of the predominant Leu4 domain. This suggests that the extensive NRPS module dynamics have diversified this family.

## MATERIALS AND METHODS

### Retrieval of LP BGCs from Pseudomonas genomes.

The conservation of BGC organization and NRPS architectural features for LP families in Pseudomonas (19) facilitated identification and delineation of such clusters in GenBank (NCBI; data as available in September 2021). We restricted further analysis to the LP systems typically absent from phytopathogens such as P. syringae since the Mycin and Peptin families (known and predicted members) were comprehensively covered in a previous study (16). Characterized BGCs of known LPs were retrieved either as a single cluster or as two unlinked clusters (Table S3). Additional BGCs were identified in genomic sequences released after the reported structural elucidation of pseudophomin (BRG-100; [67, 68]), viscosin (BBc6R8; [63]), and WLIP (PB-St2; [62, 69]). As part of a phylogenomic study (25), BGC sequences were revealed for previously described producers of WLIP (COW40, COR54; [44]) or xantholysin (COW39, COW77; [44]). In a study on LP transporter genes (19), we reported draft genomic sequences comprising LP BGCs for original producers of viscosinamide (DR54; [70]), amphisin (DSS73; [71]), and PPZPM (Wu6; [72]). For the current study, we expanded this LP BGC sequence data set for strains producing viscosin (SH10-3B; [73]), pseudophomin (P867; [74]), xantholysin (COR22, COR51; [44]), and bananamide A to C (B1M1–15), bananamide D to G (B1M1–1, B1M3–32, B2M1–30), cocoyamide (B1M2–19) (75). This collection, supplemented with available data for syringafactin, cichofactin, and virginiafactin as Factin family representatives (16), was used to compile a comprehensive pseudomonad LP-dedicated database (https://rhizoclip.be/knowledgebase/) of NRPS and A domain sequences with experimentally determined substrate specificity. Using representative NRPS sequences of the different LP families as queries, additional Pseudomonas BGCs encoding homologous enzymes were searched for in GenBank via BLASTP. Clusters with (a) fragmented NRPS gene(s) or apparent frameshift(s) were excluded. Assisted by antiSMASH analysis (bacterial version; https://antismash.secondarymetabolites.org; [76]), the clusters were inspected for characteristic synteny of regulatory and transport genes flanking predicted NRPS genes, combined with verification of the diagnostic NRPS features. Separate epimerization domains are absent from the target Pseudomonas LP systems, but they are present in NRPSs synthesizing pseudomonad pyoverdine siderophores (77) or lipopeptidic siderophores (78). Unlike these abundant siderophore and Mycin synthetases, all equipped with a single thioesterase (TE) domain, the targeted terminating NRPSs carry a tandem of two distinct TE domains (2, 13). Such tandem configuration has been reported in only few other proteobacterial NRPS systems (79–84). The presence of NRPS-flanking accessory genes facilitated the identification of NRPS systems encoded in two unconnected genomic regions. Upstream of the initiating NRPS gene, the complementary strand carries a gene encoding a dedicated LuxR-family regulatory protein of the subtype without autoinducer-binding domain (21), typically followed by a gene encoding an outer membrane protein (PleC). The latter is part of a cognate tripartite transenvelope LP exporter, together with a periplasmic adaptor protein (PleA) and an inner membrane ATP-dependent transporter (PleB) of the MacB superfamily (19, 20). The *pleAB* genes are consistently located downstream of the terminating NRPS and often accompanied by a second LuxR-family regulator gene, not strictly required for LP production. In the Tolaasin family BGCs, two accessory genes are present: an upstream transporter gene encoding a SyrD homologue (85) and a downstream aminotransferase gene enabling synthesis of 2,4-diaminobutyric acid (Dab), a nonproteinogenic AA incorporated in these CLPs (86).

10.1128/msystems.00988-22.10 TABLE S3 LP BGC accession numbers and references. Family names are denoted with capital first letter (Viscosin, Amphisin, etc.) and individual member names without capital (viscosin, amphisin, etc.). Download Table S3, PDF file, 0.2 MB. Copyright © 2023 Cesa-Luna et al. 2023 Cesa-Luna et al. https://creativecommons.org/licenses/by/4.0/ This content is distributed under the terms of the Creative Commons Attribution 4.0 International license.

### Taxonomic affiliation by *rpoD* phylogeny.

Taxonomic comparison of LP producers and type strains of related Pseudomonas species was based on *rpoD* phylogeny according to Girard et al. 2020 (87). Complete *rpoD* gene sequences were retrieved from GenBank, if available (NCBI; see Table S4 at https://doi.org/10.48804/KKFWS9). Multiple alignment was performed with MUSCLE (v3.8.425) (88). Maximum Likelihood trees were inferred with IQ-TREE (v2.1.3) (89) using the GTR+G+I model and 1,000 ultrafast bootstraps (90).

### Comparative analysis of NRPS systems per LP family.

The presumed familial relationship of a particular NRPS system, initially inferred from its global architecture (number of modules and their distribution across the NRPSs), was verified by phylogeny based on multiple AA sequence alignment of the collinearly concatenated NRPS sequences with those of known family representatives. AA sequences were aligned using MUSCLE (v3.8.425) (88), trimmed to remove short nonaligned ends, if any, and the alignment used to construct a phylogenetic tree with IQ-TREE (v2.1.3) (89) using the JTT+F+I+G model and 1,000 ultrafast bootstraps (90). NRPS domains were delineated and extracted with the NRPS-PKS tool [http://nrps.igs.umaryland.edu] (91).

### Prediction of putative LP peptide sequences based on A domain phylogeny.

Phylogenetic comparison of inspected A domains with the data set of A domains of known specificity and assumption of collinearity of domain order with sequential AA incorporation, enabled predictions of the expected peptide sequence, as described previously (48). This indicated whether a scrutinized NRPS system would probably generate one of the known peptide moieties, a potential new peptidic variant, or a divergent AA sequence. In these analyses the A domains of the LLP thanafactin behaved consistently as a homogeneous outgroup to all other LP sequences and were analyzed separately. Restriction to A domains for our comprehensive comparative analyses alleviated the need to account for the three different types of C domains and their relative positions in the NRPSs. Phylogenetic differentiation of C and C/E domains is straightforward but has limitations for stereochemical predictions, as the expected epimerization activity of a C/E domain is regularly lacking (8, 24). Therefore, predictions of AA stereochemistry were not attempted in this study. TE and C_s_ domain phylogenies are typically reflecting producer taxonomic affiliations but have poor discriminatory value for, respectively, the cyclization pattern (carboxyterminal AA linked to an internal Ser or Thr in CLPs) and the nature of the attached FA(s) (16). For selected isolates, obtained from culture collections or contacted research groups, validation of predictions was sought by chemical structure elucidation.

### Chemical characterization of LPs.

A total of 25 strains were selected for LP characterization. Culture conditions were optimized for the LP production (see Table S5 at https://doi.org/10.48804/KKFWS9). Two liters of culture was prepared for each strain, and crude extracts were obtained following a previously established protocol (19, 44, 59). The resulting crude extracts were later purified using semipreparative HPLC as previously described (19). All NMR measurements (see SData1 at https://doi.org/10.48804/KKFWS9) were performed on either a Bruker Avance III spectrometer operating at a respective ^1^H and ^13^C frequency of 500.13 MHz and 125.76 MHz and equipped with a BBI-Z probe or a Bruker Avance II spectrometer operating at a ^1^H and ^13^C frequency of 700.13 MHz and 176.05 MHz, respectively, and equipped with a 5-mm Prodigy TCI probe. The sample temperature was set to 298.0 K. Standard pulse sequences as present in the Bruker library were used throughout. High precision 5-mm NMR tubes (Norell, Landisville, NJ) were used. Dimethylformamide-d7 (DMF) (99.50%) was used as solvent throughout and was purchased from Eurisotop (Saint-Aubin, France). ^1^H and ^13^C chemical shift scales were calibrated by using the residual solvent signal using TMS as secondary reference. For NMR spectral matching, the recorded position of the CHα resonances in the ^1^H-^13^C HSQC spectra are compared with those of reference compounds with known structure/stereochemistry. Reference spectra can be obtained via the Rhizoclip website (https://www.rhizoclip.be), recorded under identical conditions.

2D spectra measured for structure elucidation included a 2D ^1^H-^1^H gCOSY, 2D ^1^H-^1^H TOCSY with a 90 ms MLEV-17 spinlock, 2D ^1^H-^1^H NOESYs with various mixing times, 2D ^1^H-^1^H off-resonance ROESYs with a mixing time of 200 ms and gradient-selected ^1^H–{^13^C} gHSQC and ^1^H–{^13^C} gHMBC. Typically, 2,048 data points were sampled in the direct dimension for 512 data points in the indirect dimension, with the spectral width set to 11 ppm and 110 ppm along the ^1^H and ^13^C dimension, respectively. The ^1^H-^13^C HMBC was measured with a 210 ppm ^13^C spectral width. For 2D processing, the spectra were zero filled to a 2,048 × 2,048 real data matrix. Before Fourier transformation, all spectra were multiplied with a squared cosine bell function in both dimensions or sine bell in the direct dimension for the gHMBC, the latter prior to magnitude calculation.

### Data availability.

Accession numbers for new nucleotide sequences are included in Table S3 and can be accessed from the NCBI BioProjects PRJNA877629 and PRJNA870320. Supplemental data files are available at https://doi.org/10.48804/KKFWS9.
