# Supplementary material for: Charting the Lipopeptidome of Nonpathogenic Pseudomonas
Source: mSystems. 2023 Jan 31;8(1):e00988-22. doi: 10.1128/msystems.00988-22 (PMC9948697; doi:10.1128/msystems.00988-22)
Supplement: TABLE S1 [file msystems.00988-22-s0008.pdf]

Table S1. Family classification and chemical structures of LPs from non-pathogenic isolates. Per family, the characteristic combination of peptide length (l) and macrocycle size (m) is indicated as [l:m]. For peptides with resolved stereochemistry the amino acid (AA) configuration is specified. The AAs involved in the macrocycle, if present, are shaded. The position and extent of this ring structure is marked by a green bar. Family names are denoted with capital first letter (Viscosin, …) and individual member names without capital (viscosin, …). BGC accession numbers and references are indicated in Table S3.

Family	Fatty acid	Amino acid by position																	
		1	2	3	4	5	6	7	8	9	10	11	12	13	14	15	16	17	18
**Bananamide [8:6]**																			
bananamide A/B/C	$C_{10/12/12:1}$OH	Leu	Asp	Thr	Leu	Leu	Gln	Leu	Ile										
MDN-0066	C_{10}OH	D-Leu	D-Glu	D-aThr	L-Leu	D-Leu	D-Ser	L-Leu	L-Ile										
prosekin	C_{10}OH	Leu	Glu	Thr	Leu	Leu	Ser	Ile	Ile										
bananamide F/E/D	$C_{10/12/12:1}$OH	Leu	Asp	Thr	Leu	Leu	Ser	Leu	Ile										
bananamide G	$C_{12\Delta}$OH	Leu	Asp	Thr	Leu	Leu	Ser	Leu	Val										
**Viscosin [9:7]**																			
viscosin	C_{10}OH	L-Leu	D-Glu	D-aThr	D-Val	L-Leu	D-Ser	L-Leu	D-Ser	L-Ile									
viscosinamide	C_{10}OH	L-Leu	D-Gln	D-aThr	D-Val	L-Leu	D-Ser	L-Leu	D-Ser	L-Ile									
WLIP	C_{10}OH	L-Leu	D-Glu	D-aThr	D-Val	D-Leu	D-Ser	L-Leu	D-Ser	L-Ile									
massetolide A/B/C	$C_{10/11/12}$OH	L-Leu	D-Glu	D-aThr	D-alle	L-Leu	D-Ser	L-Leu	D-Ser	L-Ile									
massetolide D	C_{10}OH	L-Leu	D-Glu	D-aThr	D-alle	L-Leu	D-Ser	L-Leu	D-Ser	L-Leu									
massetolide E	C_{10}OH	L-Leu	D-Glu	D-aThr	D-Val	L-Leu	D-Ser	L-Leu	D-Ser	L-Val									
massetolide F	C_{10}OH	L-Leu	D-Glu	D-aThr	D-Val	L-Leu	D-Ser	L-Leu	D-Ser	L-Leu									
massetolide G/H	$C_{11/12}$OH	L-Leu	D-Glu	D-aThr	D-Val	L-Leu	D-Ser	L-Leu	D-Ser	L-Ile									
pseudophomin A/B	$C_{10/12}$OH	L-Leu	D-Glu	D-aThr	D-Ile	D-Leu	D-Ser	L-Leu	D-Ser	L-Ile									
pseudodesmin A	C_{10}OH	L-Leu	D-Gln	D-aThr	D-Val	D-Leu	D-Ser	L-Leu	D-Ser	L-Ile									
pseudodesmin B	C_{10}OH	L-Leu	D-Gln	D-aThr	D-Val	D-Leu	D-Ser	L-Leu	D-Ser	L-Val									
**Orfamide [10:8]**																			
orfamide A/C	$C_{14/12}$OH	L-Leu	D-Glu	D-aThr	D-alle	D-Leu	D-Ser	L-Leu	L-Leu	D-Ser	L-Val								
orfamide B/D/E/F/G	$C_{14/12/14:1/16:1/16}$OH	L-Leu	D-Glu	D-aThr	D-Val	D-Leu	D-Ser	L-Leu	L-Leu	D-Ser	L-Val								
**Poaeamide [10:8]**																			
poaeamide A	C_{10}OH	L-Leu	D-Glu	D-aThr	D-Leu	L-Leu	D-Ser	L-Leu	L-Leu	D-Ser	L-Ile								
PPZM-1a (poaeamide B)	C_{10}OH	Leu	Glu	Thr	Ile	Leu	Ser	Leu	Leu	Ser	Ile								
PPZPM-2a	C_{10}OH	Leu	Glu	Thr	Val	Leu	Ser	Leu	Leu	Ser	Ile								
**Amphisin [11:9]**																			
amphisin	C_{10}OH	D-Leu	D-Asp	D-aThr	D-Leu	D-Leu	D-Ser	L-Leu	D-Gln	L-Leu	L-Ile	L-Asp							
tensin	C_{10}OH	D-Leu	D-Asp	D-aThr	D-Leu	D-Leu	D-Ser	L-Leu	D-Gln	L-Leu	L-Ile	L-Glu							
milkisin	C_{10}OH	Leu	Asp	Thr	Leu	Leu	Ser	Leu	Gln	Leu	Ile	Glu							
stechlisin B2/F	$C_{8/12}$OH	D-Leu	D-Asp	D-aThr	D-Leu	D-Leu	D-Ser	L-Leu	D-Gln	L-Leu	L-Ile	L-Glu							
stechlisin C3	C_{10}OH	D-Leu	D-Asp	D-aThr	D-Leu	D-Leu	D-Ser	L-Leu	D-Gln	L-Leu	L-Val	L-Glu							
stechlisin D3	C_{10}OH	D-Leu	D-Asp	D-aThr	D-Leu	D-Leu	D-Ser	L-Leu	D-Gln	L-Leu	L-Leu	L-Glu							
stechlisin E2	C_{10}OH	D-Leu	D-Glu	D-aThr	D-Leu	D-Leu	D-Ser	L-Leu	D-Gln	L-Leu	L-Ile	L-Glu							
pholipeptin	C_{10}OH	D-Leu	L-Asp	L-Thr	D-Leu	D-Leu	D-Ser	D-Leu	D-Ser	D-Leu	L-Ile	D-Asp							
lokisin	C_{10}OH	Leu	Asp	D-aThr	Leu	Leu	D-Ser	Leu	D-Ser	Leu	L-Ile	Asp							
anikasin	C_{10}OH	D-Leu	D-Asp	D-aThr	D-Leu	D-Leu	D-Ser	L-Leu	D-Ser	L-Leu	L-Ile	L-Asp							
arthrofactin A/D/C	$C_{10/12/12:1}$OH	D-Leu	D-Asp	D-aThr	D-Leu	D-Leu	D-Ser	L-Leu	D-Ser	L-Ile	L-Ile	L-Asp							
arthrofactin B	C_{10}OH	D-Leu	D-Asp	D-aThr	D-Leu	D-Leu	D-Ser	L-Leu	D-Ser	L-Ile	L-Ile	L-Glu							
oakridgin A/B/C	$C_{10/12/12:1}$OH	Leu	Asp	Thr	Val	Leu	Ser	Leu	Gln	Ile	Val	Asp							
oakridgin D/E/F	$C_{10/12/12:1}$OH	Leu	Asp	Thr	Val	Leu	Ser	Leu	Gln	Ile	Ile	Asp							
nepenthesin	C_{10}OH	Leu	Asp	Thr	Val	Leu	Gln	Leu	Ser	Ile	Ile	Asp							
**Cocoyamide [11:5]**																			
gacamide (cocoyamide)	C_{10}OH	D-Leu	D-Asp	D-Gln	D-Ile	D-Leu	D-Gln	D-Ser	L-Leu	L-Leu	D-Ser	L-Ile							
**Putisolvin [12:4]**																			
putisolvin III	C_6	Leu	Glu	Leu	Leu	Gln	Ser	Val	Leu	Ser	Leu	Val	Ser						
putisolvin IV	C_6	Leu	Glu	Leu	Leu	Gln	Ser	Val	Leu	Ser	Leu	Ile	Ser						
putisolvin V	C_6	Leu	Glu	Leu	Leu	Gln	Ser	Val	Leu	Ser	Leu	Leu	Ser						
**Asplenin [13:8]**																			
asplenin	C_{10}OH	Leu	Glu	Leu	Val	Gln	Ser	Val	Leu	Ser	Leu	Leu	Ser	Val					
**Entolysin [14:5]**																			
entolysin A	C_{10}OH	Leu	Glu	Gln	Val	Leu	Gln	Val	Leu	Gln	Ser	Val	Leu	Ser	Ile				
**Xantholysin [14:8]**																			
xantholysin A/C	$C_{10/12:1}$OH	Leu	Glu	Gln	Val	Leu	Gln	Ser	Val	Leu	Gln	Leu	Leu	Gln	Ile				
xantholysin B	C_{10}OH	Leu	Glu	Gln	Val	Leu	Gln	Ser	Val	Leu	Gln	Leu	Leu	Gln	Val				
**Tolaasin [18:5]**																			
tolaasin I (tolaasin C [18:0])	C_8OH	Dhb	D-Pro	D-Ser	D-Leu	D-Val	D-Ser	D-Leu	D-Val	L-Val	D-Gln	L-Leu	D-Val	Dhb	D-aThr	L-Ile	L-Hse	D-Dab	L-Lys
sessilin A	C_8OH	Dhb	Pro	Ser	Leu	Val	Gln	Leu	Val	Val	Gln	Leu	Val	Dhb	aThr	Ile	Hse	Dab	Lys
tolaasin II	C_8OH	Dhb	Pro	Ser	Leu	Val	Ser	Leu	Val	Val	Gln	Leu	Val	Dhb	aThr	Ile	Gly	Dab	Lys
tolaasin A	C_5(di-COOH)	Dhb	Pro	Ser	Leu	Val	Ser	Leu	Val	Val	Gln	Leu	Val	Dhb	aThr	Ile	Hse	Dab	Lys
tolaasin B/D	C_8OH	Dhb	Pro	Ser	Leu	Val	Ser	Leu	Val	Val	Gln	Leu	Val	Dhb	aThr	Val/Leu	Hse	Dab	Lys
tolaasin E	C_8OH	Dhb	Pro	Ser	Leu	Val	Ser	Leu	Val	Val	Gln	Leu	Val	Dhb	aThr	Leu	Gly	Dab	Lys
tolaasin F	C_8OH	Dhb	D-Pro	D-Ser	D-Leu	D-Val	D-Ser	D-Leu	D-Val	D-Val	D-Gln	L-Leu	D-Val	Dhb	D-aThr	L-Leu	L-Hse	D-Dab	L-Lys
**Syringafactin [8:0]**																			
syringafactin A/D	$C_{10/12}$OH	L-Leu	L-Leu	D-Gln	L-Leu	D-aThr	L-Val	D-Leu	L-Leu										
syringafactin B/E	$C_{10/12}$OH	Leu	Leu	Gln	Leu	Thr	Leu	Leu	Leu										
syringafactin C/F	$C_{10/12}$OH	L-Leu	L-Leu	D-Gln	L-Leu	D-Ser	L-Ile	D-Leu	L-Leu										
virginiafactin A/C	$C_{10/12}$OH	L-Leu	L-Leu	D-Gln	L-Leu	D-Ser	L-Val	D-Leu	L-Leu										
virginiafactin B/D	$C_{10/12}$OH	L-Leu	L-Leu	D-Gln	L-Leu	D-Ser	L-Ile	D-Leu	L-Leu										
cichofactin A/B	$C_{10/12}$OH	L-Leu	L-Leu	D-Gln	L-Leu	D-Gln	L-Val	D-Leu	L-Leu										
**Thanafactin [8:0]**																			
thanafactin A	C_{10}OH	D-Val	D-Ala	D-Gln	L-Ala	D-Val	L-Ala	L-Pro	L-Thr										
