# Supplementary material for: Charting the Lipopeptidome of Nonpathogenic Pseudomonas
Source: mSystems. 2023 Jan 31;8(1):e00988-22. doi: 10.1128/msystems.00988-22 (PMC9948697; doi:10.1128/msystems.00988-22)
Supplement: TABLE S2 [file msystems.00988-22-s0009.pdf]

Table S2. Taxonomic affiliation of co-producers of Mycin and Peptin LPs. Colored shading indicates by which *Pseudomonas* group (G) or subgroup (SG) specific LPs of the Mycin (blue) and Peptin (green) families are produced. A tentative structure has been reported for thanapeptin.

			Fluorescens G			Syringae G
**Family**	**Type**	**Lipopeptide**	Mandelii SG	Corrugata SG	Asplenii SG	
**Mycin**	9:9	syringomycin				
		syringostatin				
		syringotoxin				
		pseudomycin				
		cormycin				
		nunamycin				
		thanamycin				
**Peptin**	19:5	fuscopeptin				
		jessenipeptin				
	22:5	corpeptin				
		cichopeptin				
		nunapeptin				
		thanapeptin				
	22:0	sclerosin				
	22:8	syringopeptin SP22				
	25:8	syringopeptin SP25				
