# Supplementary material for: Charting the Lipopeptidome of Nonpathogenic Pseudomonas
Source: mSystems. 2023 Jan 31;8(1):e00988-22. doi: 10.1128/msystems.00988-22 (PMC9948697; doi:10.1128/msystems.00988-22)
Supplement: FIG S1 [file msystems.00988-22-s0001.pdf]

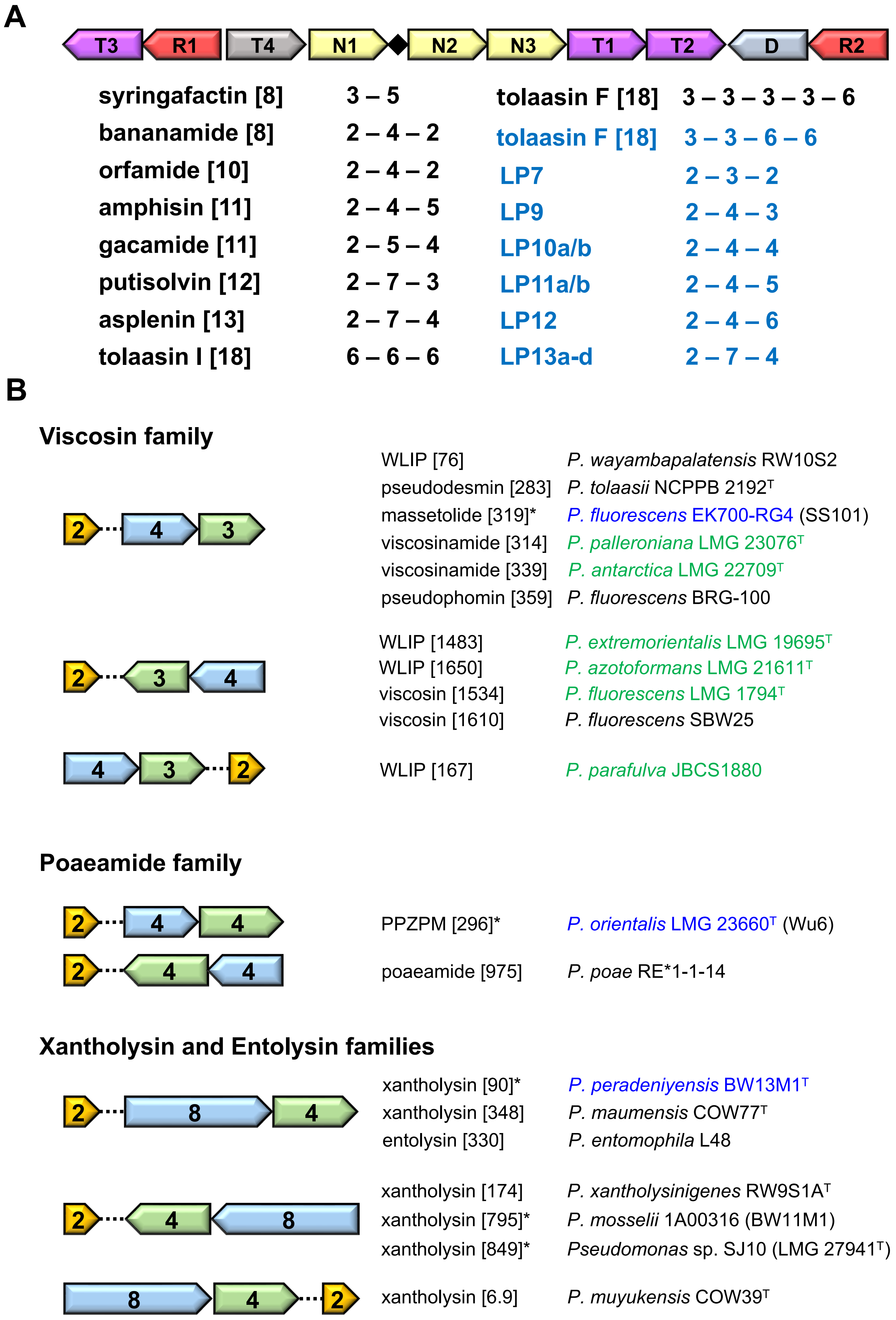

A
T3
R1
T4
N1
N2
N3
T1
T2
D
R2
syringafactin [8] 3 – 5
bananamide [8] 2 – 4 – 2
orfamide [10] 2 – 4 – 2
amphisin [11] 2 – 4 – 5
gacamide [11] 2 – 5 – 4
putisolvin [12] 2 – 7 – 3
asplenin [13] 2 – 7 – 4
tolaasin I [18] 6 – 6 – 6
tolaasin F [18] 3 – 3 – 3 – 3 – 6
tolaasin F [18] 3 – 3 – 6 – 6
LP7 2 – 3 – 2
LP9 2 – 4 – 3
LP10a/b 2 – 4 – 4
LP11a/b 2 – 4 – 5
LP12 2 – 4 – 6
LP13a-d 2 – 7 – 4
B
Viscosin family
2 4 3
WLIP [76] P. wayambapalatensis RW10S2
pseudodesmin [283] P. tolaasii NCPPB 2192T
massetolide [319]* P. fluorescens EK700-RG4 (SS101)
viscosinamide [314] P. palleroniana LMG 23076T
viscosinamide [339] P. antarctica LMG 22709T
pseudophomin [359] P. fluorescens BRG-100
2 3 4
WLIP [1483] P. extremorientalis LMG 19695T
WLIP [1650] P. azotoformans LMG 21611T
viscosin [1534] P. fluorescens LMG 1794T
viscosin [1610] P. fluorescens SBW25
4 3 2
WLIP [167] P. parafulva JBCS1880
Poaeamide family
2 4 4
PPZPM [296]* P. orientalis LMG 23660T (Wu6)
2 4 4
poaeamide [975] P. poae RE*1-1-14
Xantholysin and Entolysin families
2 8 4
xantholysin [90]* P. peradeniyensis BW13M1T
xantholysin [348] P. maumensis COW77T
entolysin [330] P. entomophila L48
2 4 8
xantholysin [174] P. xantholysinigenes RW9S1AT
xantholysin [795]* P. mosselii 1A00316 (BW11M1)
xantholysin [849]* Pseudomonas sp. SJ10 (LMG 27941T)
8 4 2
xantholysin [6.9] P. muyukensis COW39T
