# Supplementary figures and images for: Charting the Lipopeptidome of Nonpathogenic Pseudomonas
Source: mSystems. 2023 Jan 31;8(1):e00988-22. doi: 10.1128/msystems.00988-22 (PMC9948697; doi:10.1128/msystems.00988-22)

# A massetolide

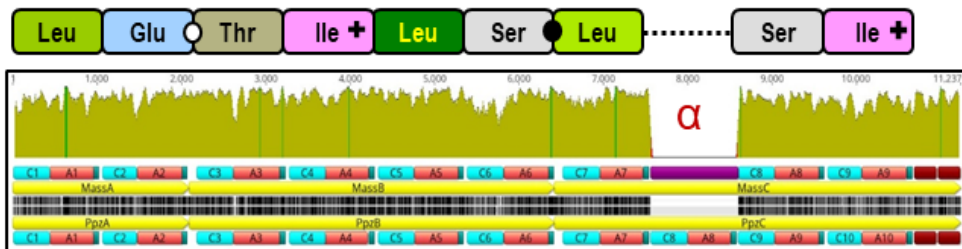

PPZPM

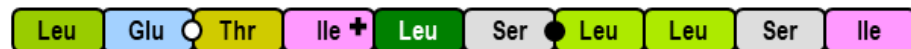

# B poaeamide A

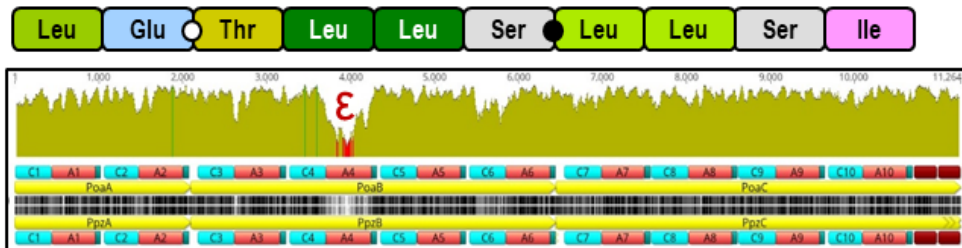

PPZPM

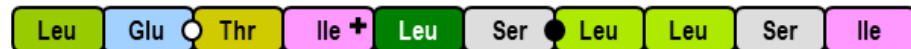

Supplement: FIG S5 [file msystems.00988-22-s0005.pdf]

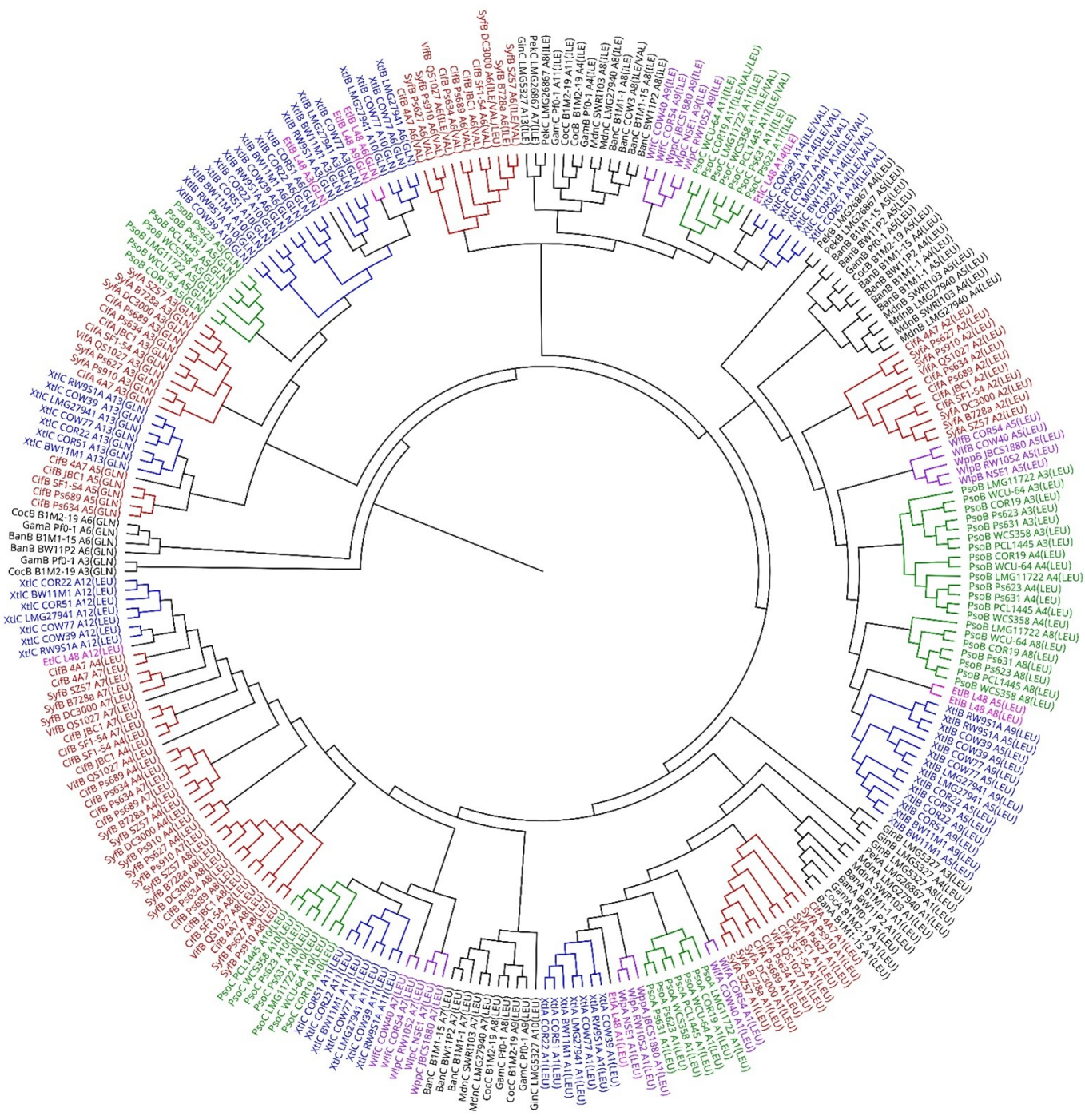

Supplement: FIG S4 [file msystems.00988-22-s0004.pdf]

**A** LP7

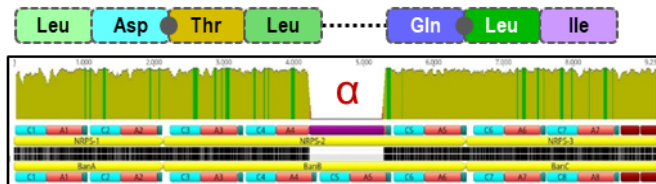

bananamide A-C

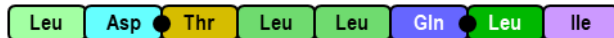

**B** bananamide A-C

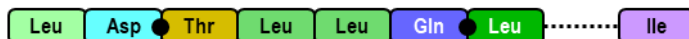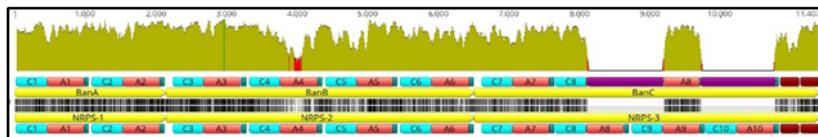

LP10a

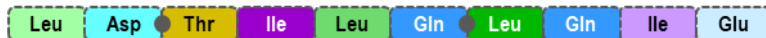

**C** LP10a

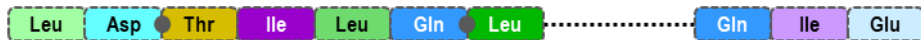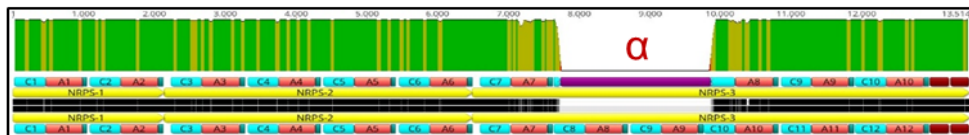

LP12

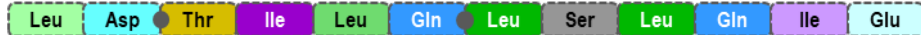

Supplement: FIG S6 [file msystems.00988-22-s0006.pdf]

LP9

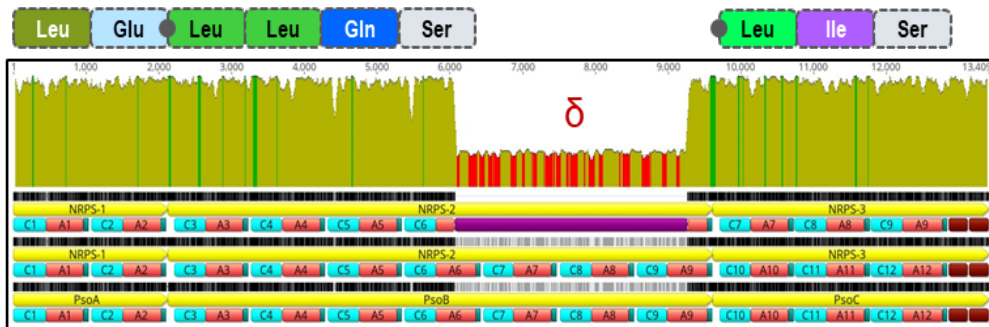

putisolvin

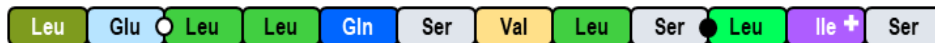

LP10b

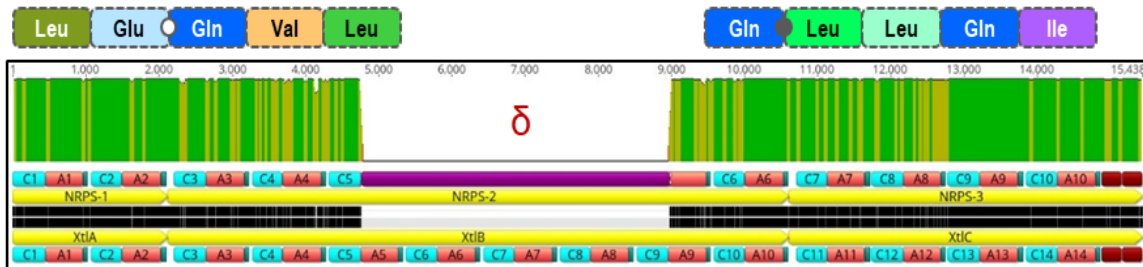

xantholysin

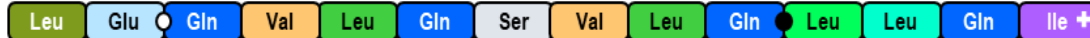

Supplement: FIG S7 [file msystems.00988-22-s0007.pdf]
