# Supplementary material for: Charting the Lipopeptidome of Nonpathogenic Pseudomonas
Source: mSystems. 2023 Jan 31;8(1):e00988-22. doi: 10.1128/msystems.00988-22 (PMC9948697; doi:10.1128/msystems.00988-22)
Supplement: FIG S3 [file msystems.00988-22-s0003.pdf]

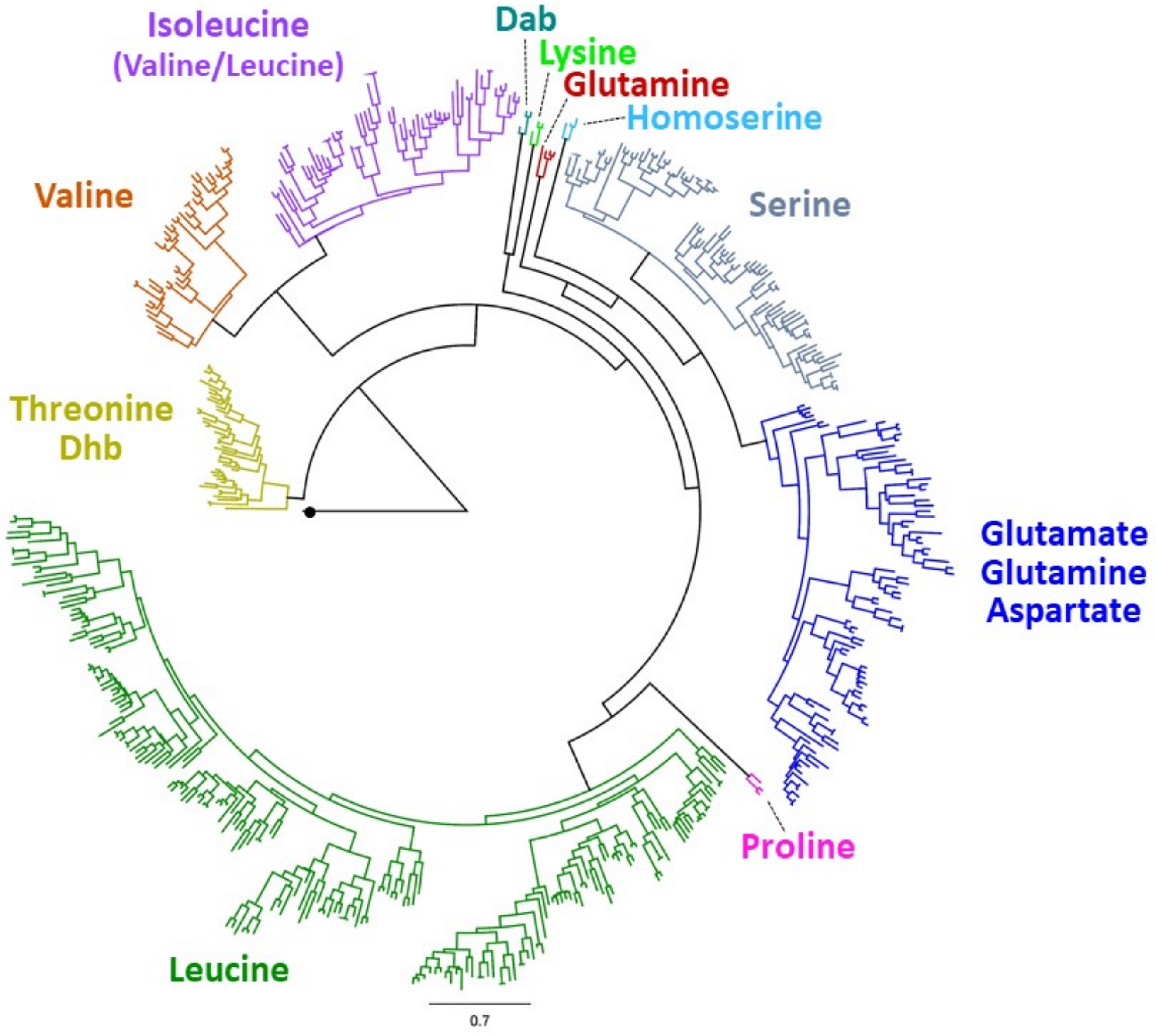
Isoleucine
(Valine/Leucine)
Dab
Lysine
Glutamine
Homoserine
Valine
Serine
Threonine
Dhb
Glutamate
Glutamine
Aspartate
Proline
Leucine
0.7
