# Supplementary material for: Charting the Lipopeptidome of Nonpathogenic Pseudomonas
Source: mSystems. 2023 Jan 31;8(1):e00988-22. doi: 10.1128/msystems.00988-22 (PMC9948697; doi:10.1128/msystems.00988-22)
Supplement: TABLE S3 [file msystems.00988-22-s0010.pdf]

Table S3. LP BGC accession numbers and references. Family names are denoted with capital first letter (Viscosin, Amphisin,…) and individual member names without capital (viscosin, amphisin, …).

Lipopeptide	NRPS system	Species	Strain	Accession nr (BGC unsplitted)	Accession nr (BGC splitted)		Reference
					A-region	BC-region	
amphisin	Ams	*P. fluorescens*	DSS73	JAFLXG010000010			(1)
anikasin	Ani	*P. fluorescens*	HKI0770	LVEJ01000013			(2)
	Ani	*P. fluorescens*	Ps655	CABVHJ010000006			(3)
	Ani	*P. fluorescens*	Ps925	CABVJG010000015			(3)
arthrofactin	Arf	*Pseudomonas* sp.	MIS38	AB107223			(4)
asplenin	Asp	*P. fuscovaginae*	UPB0736	CP100603			(5)
bananamide (A-C)	Ban	*P. bananamidigenes*	BW11P2^T	KX437753			(6)
	Ban	*P. koreensis*	B1M1-15	JAOEJU010000008			(7); this study
bananamide (D-G)	Ban	*P. botevensis*	COW3^T	MN480426			(8)
	Ban	*P. koreensis*	B1M3-32	JAOSKY000000000			(7); this study
	Ban	*Pseudomonas* sp.	B1M1-1	JAOSKZ000000000			(7); this study
	Ban	*Pseudomonas* sp.	B2M1-30	JAOEJT010000008			(7); this study
cichofactin A/B	Cif	*P. cichorii*	JBC1	CP007039			(9)
		P. cichorii	SF1-54	KJ513093			(10)
		Pseudomonas sp.	Ps634	CABVHA010000009			(3)
		Pseudomonas sp.	Ps689	CABVIA010000014			(3)
cocoyamide	Coc	*Pseudomonas* sp.	COW5	JAPHVP000000000			(11)
	Coc	*P. koreensis*	B1M2-19	JAOEJS000000000			(7); this study
entolysin	Etl	*P. entomophila*	L48^T		CT573326	CT573326	(12)
gacamide	Gam	*P. fluorescens*	Pf0-1	CP000094			(13)
lokisin	Lok	*Pseudomonas* sp.	COR10	MK534107			(11)
massetolide	Mass	*P. lactis*	SS101		EU199080	EU199081	(14)
MA026 (xantholysin)	Xtl	*Pseudomonas* sp.	RtIB026		AP023348	AP023348	(15)
MDN-0066	Mdn	*P. granadensis*	LMG 27940^T	LT629778			(16)
		P. azadiae	SWRI103^T	JABBCM010000002			(17)
milkisin	Mlk	*P. crudilactis*	UCMA 17988^T	WXVV01000014			(18)
orfamide	Ofa	*P. aestus*	CMR5c	KT613918			(17, 19)
		P. protegens	CHA0^T	CP003190			(20)
		P. protegens	Pf-5	CP000076			(17, 21)
		P. sessilinigenes	CMR12a^T	JQ309921; CP027706			(17, 22)
		Pseudomonas sp.	PH1b	JAAARL010000025			(17)
poaeamide A	Poa	*P. poae*	RE*1-1-14		CP004045	CP004045	(23)
poaeamide B	Ppz	*P. synxantha*	CR32		KU936045	KU936046	(6)
PPZPM	Ppz	*Pseudomonas* sp.	Wu6		JAFLXH010000010	JAFLXH010000004	(24, 25)

Table S3 Continued

prosekin	Pek	*P. prosekii*	LMG 26867^{T}	LT629762			(26)
pseudodesmin	Pdm	*Pseudomonas* sp.	COR52	MT577358			(27)
pseudodesmin	Pse	*P. tolaasii*	NCPPB 2192^{T}		PHHD01000001	PHHD01000001	(28)
pseudophomin	Pmn	*Pseudomonas* sp.	BRG-100				(29, 30)
		Pseudomonas sp.	P867				(31), this study
putisolvin	Pso	*Pseudomonas* sp.	COR19	MT511055			(32)
		P. putida	PCL1445	DQ151887			(33)
		P. fluorescens	Ps623	CABVHC010000010			(3)
		P. fluorescens	Ps631	CABVHF010000003			(3)
		P. fulva	LMG 11722^{T}	BBIQ01000007			(26)
		P. vlassakiae	WCU_60, WCU_64	MT511056, MT511054			(32)
sessilin	Ses	*P. sessilinigenes*	CMR12a^{T}	CP027706			(22)
stechlisin/tensin	Ste	*Pseudomonas* sp.	FhG1000052	MT080808			(34)
syringafactin	Syf	*P. syringae pv. syringae*	B728a	CP000075			(35)
		P. syringae pv. *tomato*	DC3000	AE016853			(36)
		Pseudomonas sp.	SZ57	MQQ37657			(37)
		P. putida	Ps627	CAJVQG010000001			(3)
		P. putida	Ps910	CABVIX010000005			(3)
tensin	Ten	*P. zeae*	OE 48.2^{T}	CP077090			(25)
tensin-like	Ten	*P. fluorescens*	Ps619	CABVHD010000011			(3)
		P. fluorescens	Ps681	CABVGV010000041			(3)
		P. fluorescens	Ps684	CABVHM010000001			(3)
		P. fluorescens	Ps896	CABVIN010000007			(3)
		P. fluorescens	Ps941	CABVJC010000014			(3)
		P. fluorescens	Ps947	CABVJI010000006			(3)
thanafactin	Tha	*P. fluorescens*	DSM 11579	JAAOIQ010000001			(38)
		Pseudomonas sp.	SH-C52	MT431590			(38)
tolaasin I/II/B/D/E	Tol	*P. tolaasii*	NCPPB 2192^{T}	PHHD01000001			(28)
tolaasin F	Taa	*P. costantinii*	DSM 16734^{T} (LMG 22119^{T})	HE967327			(39)
virginiafactin A/B/C	Vif	*Pseudomonas* sp.	QS1027	PHSU01000004			(9)
viscosin	Visc	*P. fluorescens*	SBW25		AM181176	AM181176	(40)
		P. fluorescens	SH10-3B		JAFLXD010000003	JAFLXD010000056	(41); this study
		P. fluorescens	Ps664		CABVGU010000024	CABVGU010000006	(3)
		P. fluorescens	Ps687		CABVHN010000016	CABVHN010000020	(3)
		P. fluorescens	Ps720		CABVHZ010000031	CABVHZ010000021	(3)
	Viy	*Pseudomonas* sp.	BBc6R8	AKXH02000048 & AKXH02000047			(42); Deveau and Gross, person. commun.

Table S3 Continued

viscosinamide	Vsa	*Pseudomonas* sp.	ICBG1301		JAEKGB010000001	JAEKGB010000009	(43)
	Vsm	*P. carnis*	DR54		JAFLXE010000039	JAFLXE010000037	(26, 44)
	Vsm	*Pseudomonas* sp.	A2W4.9		MT749674	MT771985	(27)
WLIP	Wip	*P. fluorescens*	LMG 5329		JQ974025	JQ974026	(45)
		P. fluorescens	Ps663		CABVGT010000002	CABVGT010000005	(3)
		P. fluorescens	Ps682		CABVGW010000011	CABVGW010000014	(3)
		P. fluorescens	Ps683		LR700640	LR700645	(3)
		P. fluorescens	Ps907		CABVIZ010000001	CABVIZ010000004	(3)
	Wlc	*P. chlororaphis*	PB-St2	CP027716			(46, 47)
	Wlf	*P. fakonensis*	COW40^T	CP077076			(11, 25)
	Wlf	*P. xanthosomae*	COR54^T	CP077075			(11, 25)
	Wlp	*P. wayambapalatensis*	RW10S2		JN982332	JN982333	(48)
	Wlp	*Pseudomonas* sp.	NSE1		MK534106	MK650230	(27)
xantholysin	Xtl	*P. mosselii*	BW11M1		KC297505	KC297506	(17, 49)
		P. maumuensis	COW77^T		CP077077	CP077077	(11, 25)
		P. muyukensis	COW39^T		CP077073	CP077073	(11, 25)
		P. soli	LMG 27941^T		FOEQ01000005	FOEQ01000004/28/11	(50); this study (resequencing and assembly *xtlBC* contigs)
		P. peradeniyensis	COR22		JAOSLB010000025	JAOSLB010000028	(51); this study
		P. peradeniyensis	COR51		JAOSLA010000032	JAOSLA010000033	(11); this study
		P. xantholysinigenes	RW9S1A^T		CP077095	CP077095	(26)

Supplementary References

1. Sørensen D, Nielsen TH, Christophersen C, Sørensen J, Gajhede M. 2001. Cyclic lipoundecapeptide amphisin from *Pseudomonas* sp. strain DSS73. Acta Crystallogr C 57:1123–1124.

2. Götze S, Herbst-Irmer R, Klapper M, Görls H, Schneider KRA, Barnett R, Burks T, Neu U, Stallforth P. 2017. Structure, Biosynthesis, and Biological Activity of the Cyclic Lipopeptide Anikasin. ACS Chem Biol 12:2498–2502.

3. Pacheco-Moreno A, Stefanato FL, Ford JJ, Trippel C, Uszkoreit S, Ferrafiat L, Grenga L, Dickens R, Kelly N, Kingdon AD, Ambrosetti L, Nepogodiev SA, Findlay KC, Cheema J, Trick M, Chandra G, Tomalin G, Malone JG, Truman AW. 2021. Pan-genome analysis identifies intersecting roles for *Pseudomonas* specialized metabolites in potato pathogen inhibition. eLife 10:e71900.

4. Roongsawang N, Hase K ichi, Haruki M, Imanaka T, Morikawa M, Kanaya S. 2003. Cloning and characterization of the gene cluster encoding arthrofactin synthetase from *Pseudomonas* sp. MIS38. Chem Biol 10:869–880.

5. Ferrarini E, Špacapan M, Lam VB, McCann A, Cesa-Luna C, Marahatta BP, De Pauw E, De Mot R, Venturi V, Höfte M. 2022. Versatile role of *Pseudomonas fuscovaginae* cyclic lipopeptides in plant and microbial interactions. Frontiers in Plant Science 13:1008980.

6. Nguyen DD, Melnik AV, Koyama N, Lu X, Schorn M, Fang J, Aguinaldo K, Lincecum TL, Ghequire MGK, Carrion VJ, Cheng TL, Duggan BM, Malone JG, Mauchline TH, Sanchez LM, Kilpatrick AM, Raaijmakers JM, De Mot R, Moore BS, Medema MH, Dorrestein PC. 2016. Indexing the *Pseudomonas* specialized metabolome enabled the discovery of poaeamide B and the bananamides. Nature Microbiology 2:1–10.

7. Omoboye OO. 2019. Cyclic lipopeptide diversity and biocontrol versatility of *Pseudomonas* species associated with the cocoyam rhizosphere. PhD Thesis. Ghent University, Ghent, Belgium.

8. Omoboye OO, Geudens N, Duban M, Chevalier M, Flahaut C, Martins JC, Leclère V, Oni FE, Höfte M. 2019. *Pseudomonas* sp. COW3 Produces New Bananamide-Type Cyclic Lipopeptides with Antimicrobial Activity against *Pythium myriotylum* and *Pyricularia oryzae*. Molecules 24:4170–4170.

9. Götze S, Arp J, Lackner G, Zhang S, Kries H, Klapper M, García-Altares M, Willing K, Günther M, Stallforth P. 2019. Structure elucidation of the syringafactin lipopeptides provides insight in the evolution of nonribosomal peptide synthetases. Chem Sci 10:10979–10990.

10. Pauwelyn E, Huang C-J, Ongena M, Leclère V, Jacques P, Bleyaert P, Budzikiewicz H, Schäfer M, Höfte M. 2013. New linear lipopeptides produced by *Pseudomonas cichorii* SF1-54 are involved in virulence, swarming motility, and biofilm formation. 5. Molecular Plant-Microbe Interactions 26:585–598.

11. Oni FE, Geudens N, Omoboye OO, Bertier L, Hua HGK, Adiobo A, Sinnaeve D, Martins JC, Höfte M. 2019. Fluorescent *Pseudomonas* and cyclic lipopeptide diversity in the rhizosphere of cocoyam (*Xanthosoma sagittifolium*). Environ Microbiol 21:1019–1034.

12. Vallet-Gely I, Novikov A, Augusto L, Liehl P, Bolbach G, Péchy-Tarr M, Cosson P, Keel C, Caroff M, Lemaitre B. 2010. Association of hemolytic activity of *Pseudomonas entomophila*, a versatile soil bacterium, with cyclic lipopeptide production. Appl Environ Microbiol 76:910–921.

13. Jahanshah G, Yan Q, Gerhardt H, Pataj Z, Lämmerhofer M, Pianet I, Josten M, Sahl HG, Silby MW, Loper JE, Gross H. 2019. Discovery of the cyclic lipopeptide gacamide a by genome mining and repair of the defective gaca regulator in *Pseudomonas fluorescens* pf0-1. Journal of Natural Products 82:301–308.

14. De Bruijn I, De Kock MJD, De Waard P, Van Beek TA, Raaijmakers JM. 2008. Massetolide A biosynthesis in *Pseudomonas fluorescens*. J Bacteriol 190:2777–2789.

15. Uchiyama C, Fukuda A, Mukaiyama M, Nakazawa Y, Kuramochi Y, Muguruma K, Arimoto M, Ninomiya A, Kako K, Katsuyama Y, Konno S, Taguchi A, Takayama K, Taniguchi A, Nagumo Y, Usui T, Hayashi Y. 2021. Structural Revision of Natural Cyclic Depsipeptide MA026 Established by Total Synthesis and Biosynthetic Gene Cluster Analysis. Angewandte Chemie International Edition 60:8792–8797.

16. Cautain B, Pedro N de, Schulz C, Pascual J, Sousa T da S, Martin J, Pérez-Victoria I, Asensio F, González I, Bills GF, Reyes F, Genilloud O, Vicente F. 2015. Identification of the Lipodepsipeptide MDN-0066, a Novel Inhibitor of VHL/HIF Pathway Produced by a New *Pseudomonas* Species. PLOS ONE 10:e0125221.

17. De Roo V, Verleysen Y, Kovács B, De Vleeschouwer M, Muangkaew P, Girard L, Höfte M, De Mot R, Madder A, Geudens N, Martins JC. 2022. An Nuclear Magnetic Resonance Fingerprint Matching Approach for the Identification and Structural Re-Evaluation of *Pseudomonas* Lipopeptides. Microbiol Spectr 10:e0126122.

18. Schlusselhuber M, Godard J, Sebban M, Bernay B, Garon D, Seguin V, Oulyadi H, Desmasures N. 2018. Characterization of Milkisin, a Novel Lipopeptide With Antimicrobial Properties Produced By *Pseudomonas* sp. UCMA 17988 Isolated From Bovine Raw Milk. Frontiers in Microbiology 9:1030.

19. Ma Z, Hua GKH, Ongena M, Höfte M. 2016. Role of phenazines and cyclic lipopeptides produced by *Pseudomonas* sp. CMR12a in induced systemic resistance on rice and bean. Environ Microbiol Rep 8:896–904.

20. Ma Z, Geudens N, Kieu NP, Sinnaeve D, Ongena M, Martins JC, Höfte M. 2016. Biosynthesis, Chemical Structure, and Structure-Activity Relationship of Orfamide Lipopeptides Produced by *Pseudomonas protegens* and Related Species. Front Microbiol 7:382.

21. Gross H, Stockwell VO, Henkels MD, Nowak-Thompson B, Loper JE, Gerwick WH. 2007. The genomisotopic approach: a systematic method to isolate products of orphan biosynthetic gene clusters. Chem Biol 14:53–63.

22. D’aes J, Kieu NP, Léclère V, Tokarski C, Olorunleke FE, De Maeyer K, Jacques P, Höfte M, Ongena M. 2014. To settle or to move? The interplay between two classes of cyclic lipopeptides in the biocontrol strain *Pseudomonas* CMR12a. Environ Microbiol 16:2282–2300.

23. Zachow C, Jahanshah G, de Bruijn I, Song C, Ianni F, Pataj Z, Gerhardt H, Pianet I, Lämmerhofer M, Berg G, Gross H, Raaijmakers JM. 2015. The Novel Lipopeptide Poaeamide of the Endophyte *Pseudomonas poae* RE*1-1-14 Is Involved in Pathogen Suppression and Root Colonization. Mol Plant Microbe Interact 28:800–810.

24. Weißhoff H, Hentschel S, Zaspel I, Jarling R, Krause E, Pham TLH. 2014. PPZPMs - a Novel Group of Cyclic Lipodepsipeptides Produced by the *Phytophthora alni* Associated Strain *Pseudomonas* sp. JX090307 -the Missing Link between the Viscosin and Amphisin Group. Natural Product Communications 9:989–996.

25. Girard L, Lood C, Höfte M, Vandamme P, Rokni-Zadeh H, van Noort V, Lavigne R, De Mot R. 2021. The Ever-Expanding *Pseudomonas* Genus: Description of 43 New Species and Partition of the *Pseudomonas putida* Group. 8. Microorganisms 9:1766.

26. Girard L, Geudens N, Pauwels B, Höfte M, Martins JC, De Mot R. 2022. Transporter Gene-Mediated Typing for Detection and Genome Mining of Lipopeptide-Producing *Pseudomonas*. Applied and Environmental Microbiology 88:e01869-21.

27. Oni FE, Geudens N, Adiobo A, Omoboye OO, Enow EA, Onyeka JT, Salami AE, De Mot R, Martins JC, Höfte M. 2020. Biosynthesis and antimicrobial activity of pseudodesmin and viscosinamide cyclic lipopeptides produced by pseudomonads associated with the cocoyam rhizosphere. Microorganisms 8:1–26.

28. Hermenau R, Kugel S, Komor AJ, Hertweck C. 2020. Helper bacteria halt and disarm mushroom pathogens by linearizing structurally diverse cyclolipopeptides. Proc Natl Acad Sci U S A 117:23802–23806.

29. Pedras MSC, Ismail N, Quail JW, Boyetchko SM. 2003. Structure, chemistry, and biological activity of pseudophomins A and B, new cyclic lipodepsipeptides isolated from the biocontrol bacterium *Pseudomonas fluorescens*. Phytochemistry 62:1105–1114.

30. Dumonceaux TJ, Town J, Links MG, Boyetchko S. 2014. High-Quality Draft Genome Sequence of *Pseudomonas* sp. BRG100, a Strain with Bioherbicidal Properties against *Setaria viridis* (Green Foxtail) and Other Pests of Agricultural Significance. Genome Announc 2:e00995-14.

31. Reybroeck W, De Vleeschouwer M, Marchand S, Sinnaeve D, Heylen K, De Block J, Madder A, Martins JC, Heyndrickx M. 2014. Cyclic lipodepsipeptides produced by *Pseudomonas* spp. naturally present in raw milk induce inhibitory effects on microbiological inhibitor assays for antibiotic residue screening. PLoS One 9:e98266.

32. Oni FE, Geudens N, Onyeka JT, Olorunleke OF, Salami AE, Omoboye OO, Arias AA, Adiobo A, De Neve S, Ongena M, Martins JC, Höfte M. 2020. Cyclic lipopeptide-producing *Pseudomonas koreensis* group strains dominate the cocoyam rhizosphere of a *Pythium* root rot suppressive soil contrasting with *P. putida* prominence in conducive soils. Environmental Microbiology 22:5137–5155.

33. Kuiper I, Lagendijk EL, Pickford R, Derrick JP, Lamers GEM, Thomas-Oates JE, Lugtenberg BJJ, Bloemberg GV. 2004. Characterization of two *Pseudomonas putida* lipopeptide biosurfactants, putisolvin I and II, which inhibit biofilm formation and break down existing biofilms. Mol Microbiol 51:97–113.

34. Marner M, Patras MA, Kurz M, Zubeil F, Förster F, Schuler S, Bauer A, Hammann P, Vilcinskas A, Schäberle TF, Glaeser J. 2020. Molecular Networking-Guided Discovery and Characterization of Stechlisins, a Group of Cyclic Lipopeptides from a *Pseudomonas* sp. J Nat Prod 83:2607–2617.

35. Burch AY, Zeisler V, Yokota K, Schreiber L, Lindow SE. 2014. The hygroscopic biosurfactant syringafactin produced by *Pseudomonas syringae* enhances fitness on leaf surfaces during fluctuating humidity. Environmental Microbiology 16:2086–2098.

36. Berti AD, Greve NJ, Christensen QH, Thomas MG. 2007. Identification of a Biosynthetic Gene Cluster and the Six Associated Lipopeptides Involved in Swarming Motility of *Pseudomonas syringae* pv. tomato DC3000. Journal of Bacteriology 189:6312–6323.

37. Zhang S, Mukherji R, Chowdhury S, Reimer L, Stallforth P. 2021. Lipopeptide-mediated bacterial interaction enables cooperative predator defense. Proceedings of the National Academy of Sciences 118:e2013759118.

38. Kirchner N, Cano-Prieto C, Schulz-Fincke A-C, Gütschow M, Ortlieb N, Moschny J, Niedermeyer THJ, Horak J, Lämmerhofer M, van der Voort M, Raaijmakers JM, Gross H. 2021. Discovery of Thanafactin A, a Linear, Proline-Containing Octalipopeptide from *Pseudomonas* sp. SH-C52, Motivated by Genome Mining. J Nat Prod 84:101–109.

39. Scherlach K, Lackner G, Graupner K, Pidot S, Bretschneider T, Hertweck C. 2013. Biosynthesis and mass spectrometric imaging of tolaasin, the virulence factor of brown blotch mushroom disease. Chembiochem 14:2439–2443.

40. De Bruijn I, De Kock MJD, Yang M, De Waard P, Van Beek TA, Raaijmakers JM. 2007. Genome-based discovery, structure prediction and functional analysis of cyclic lipopeptide antibiotics in *Pseudomonas* species. Mol Microbiol 63:417–428.

41. Laycock MV, Hildebrand PD, Thibault P, Walter JA, Wright JLC. 1991. Viscosin, a potent peptidolipid biosurfactant and phytopathogenic mediator produced by a pectolytic strain of *Pseudomonas fluorescens*. J Agric Food Chem 39:483–489.

42. Deveau A, Gross H, Palin B, Mehnaz S, Schnepf M, Leblond P, Dorrestein PC, Aigle B. 2016. Role of secondary metabolites in the interaction between *Pseudomonas fluorescens* and soil microorganisms under iron-limited conditions. FEMS Microbiol Ecol 92:fiw107.

43. Fukuda TTH, Pereira CF, Melo WGP, Menegatti C, Andrade PHM, Groppo M, Lacava PT, Currie CR, Pupo MT. 2021. Insights Into the Ecological Role of *Pseudomonas* spp. in an Ant-plant Symbiosis. Front Microbiol 12:621274.

44. Nielsen TH, Christophersen C, Anthoni U, Sørensen J. 1999. Viscosinamide, a new cyclic depsipeptide with surfactant and antifungal properties produced by *Pseudomonas fluorescens* DR54. Journal of Applied Microbiology 87:80–90.

45. Rokni-Zadeh H, Li W, Yilma E, Sanchez-Rodriguez A, De Mot R. 2013. Distinct lipopeptide production systems for WLIP (white line-inducing principle) in *Pseudomonas fluorescens* and *Pseudomonas putida*. Environ Microbiol Rep 5:160–169.

46. Mehnaz S, Saleem RSZ, Yameen B, Pianet I, Schnakenburg G, Pietraszkiewicz H, Valeriote F, Josten M, Sahl H-G, Franzblau SG, Gross H. 2013. Lahorenoic acids A-C, ortho-dialkyl-substituted aromatic acids from the biocontrol strain *Pseudomonas aurantiaca* PB-St2. J Nat Prod 76:135–141.

47. Biessy A, Novinscak A, Blom J, Léger G, Thomashow LS, Cazorla FM, Josic D, Filion M. 2019. Diversity of phytobeneficial traits revealed by whole-genome analysis of worldwide-isolated phenazine-producing *Pseudomonas* spp. Environ Microbiol 21:437–455.

48. Rokni-Zadeh H, Li W, Sanchez-Rodriguez A, Sinnaeve D, Rozenski J, Martins JC, De Mot R. 2012. Genetic and Functional Characterization of Cyclic Lipopeptide White-Line-Inducing Principle (WLIP) Production by Rice Rhizosphere Isolate *Pseudomonas putida* RW10S2. Applied and Environmental Microbiology 78:4826–4834.

49. Li W, Rokni-Zadeh H, De Vleeschouwer M, Ghequire MGK, Sinnaeve D, Xie G-L, Rozenski J, Madder A, Martins JC, De Mot R. 2013. The Antimicrobial Compound Xantholysin Defines a New Group of *Pseudomonas* Cyclic Lipopeptides. PLoS ONE 8:e62946–e62946.

50. Pascual J, García-López M, Carmona C, Sousa T da S, de Pedro N, Cautain B, Martín J, Vicente F, Reyes F, Bills GF, Genilloud O. 2014. *Pseudomonas soli* sp. nov., a novel producer of xantholysin congeners. Syst Appl Microbiol 37:412–416.

51. Oni FE. 2017. Cyclic lipopeptides produced by *Pseudomonas* spp. associated with the cocoyam (*Xanthosoma sagittifolium* (L.) Schott) rhizosphere: diversity, regulation, secretion and biological activity. PhD Thesis. Ghent University, Ghent, Belgium.
