# Supplementary material for: Charting the Lipopeptidome of Nonpathogenic Pseudomonas
Source: mSystems. 2023 Jan 31;8(1):e00988-22. doi: 10.1128/msystems.00988-22 (PMC9948697; doi:10.1128/msystems.00988-22)
Supplement: FIG S2 [file msystems.00988-22-s0002.pdf]

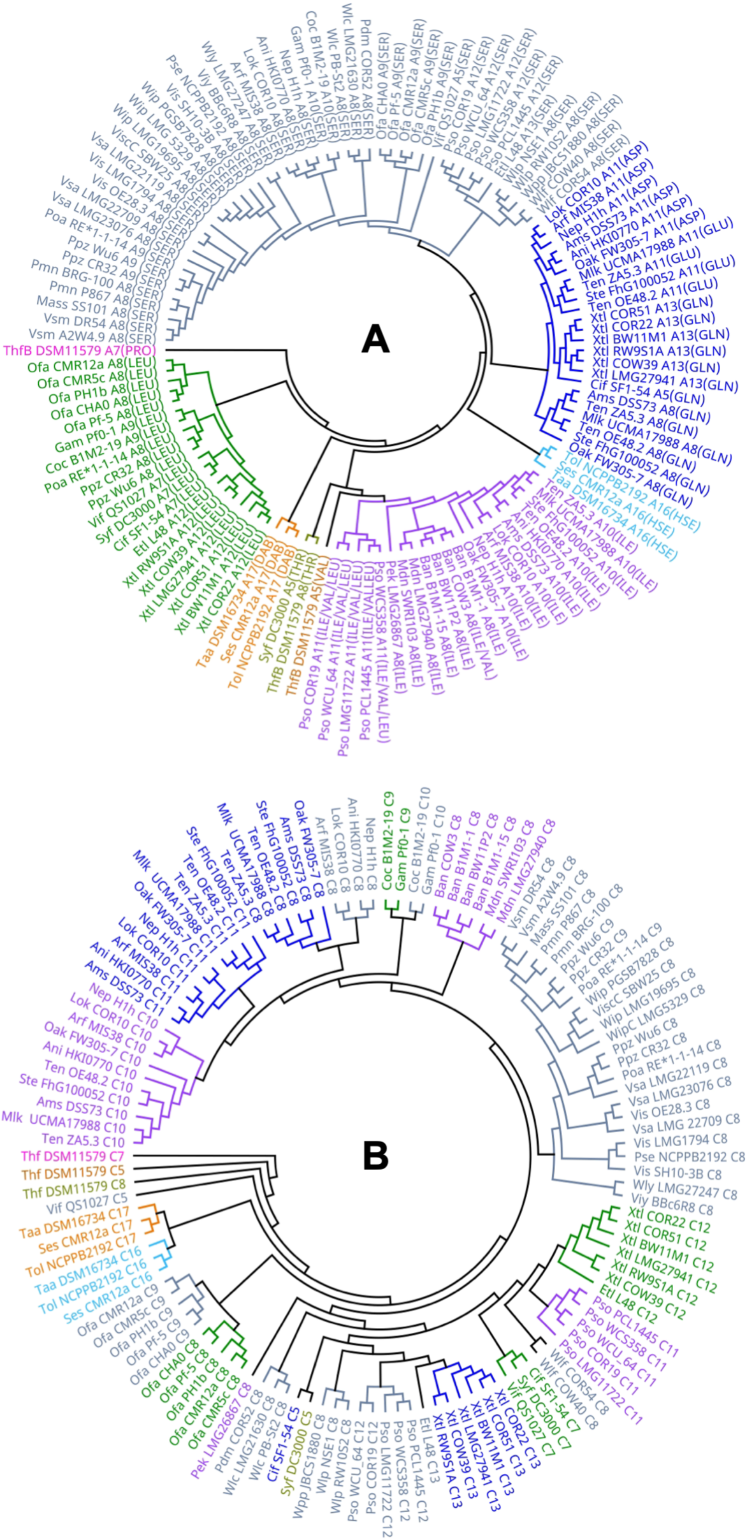
A
ThfB DSM11579 A7(PRO)
Vsm A2W4.9 A8(SER)
Vsm DR54 A8(SER)
Mass SS101 A8(SER)
Pmn P867 A8(SER)
Pmn BRG-100 A8(SER)
Ppz CR32 A9(SER)
Ppz Wu6 A9(SER)
Poa RE*1-1-14 A9(SER)
Vsa LMG23076 A8(SER)
Vsa LMG22709 A8(SER)
Vis OE28.3 A8(SER)
Vis LMG1794 A8(SER)
Vsa LMG22119 A8(SER)
ViscC SBW25 A8(SER)
Wip LMG19695 A8(SER)
Wip LMG 5329 A8(SER)
Wip PGSB7828 A8(SER)
Vis SH10-3B A8(SER)
Pse NCPPB2192 A8(SER)
Viy BBc6R8 A8(SER)
Wly LMG27247 A8(SER)
Arf MIS38 A8(SER)
Lok COR10 A8(SER)
Ani HKI0770 A8(SER)
Nep H1h A8(SER)
Gam Pf0-1 A10(SER)
Coc B1M2-19 A10(SER)
Wlc PB-St2 A8(SER)
Wlc LMG21630 A8(SER)
Pdm COR52 A8(SER)
Ofa CHA0 A9(SER)
Ofa Pf-5 A9(SER)
Ofa CMR12a A9(SER)
Ofa CMR5c A9(SER)
Ofa PH1b A9(SER)
Vif QS1027 A5(SER)
Pso COR19 A12(SER)
Pso WCU_64 A12(SER)
Pso LMG11722 A12(SER)
Pso WCS358 A12(SER)
Pso PCL1445 A12(SER)
Etl L48 A13(SER)
Wlp NSE1 A8(SER)
Wlp RW1052 A8(SER)
Wpp JBCS1880 A8(SER)
Wlf COW40 A8(SER)
Wlf COR54 A8(SER)
Lok COR10 A11(ASP)
Arf MIS38 A11(ASP)
Nep H1h A11(ASP)
Ams DSS73 A11(ASP)
Ani HKI0770 A11(ASP)
Oak FW305-7 A11(ASP)
Mlk UCMA17988 A11(GLU)
Ten ZA5.3 A11(GLU)
Ste FhG100052 A11(GLU)
Ten OE48.2 A11(GLU)
Xtl COR51 A13(GLN)
Xtl COR22 A13(GLN)
Xtl BW11M1 A13(GLN)
Xtl RW9S1A A13(GLN)
Xtl COW39 A13(GLN)
Xtl LMG27941 A13(GLN)
Cif SF1-54 A5(GLN)
Ams DSS73 A8(GLN)
Ten ZA5.3 A8(GLN)
Mlk UCMA17988 A8(GLN)
Ten OE48.2 A8(GLN)
Ste FhG100052 A8(GLN)
Oak FW305-7 A8(GLN)
Tol NCPPB2192 A16(HSE)
Ses CMR12a A16(HSE)
Taa DSM16734 A16(HSE)
Ten ZA5.3 A10(ILE)
Mlk UCMA17988 A10(ILE)
Ste FhG100052 A10(ILE)
Ten OE48.2 A10(ILE)
Ams DSS73 A10(ILE)
Ani HKI0770 A10(ILE)
Lok COR10 A10(ILE)
Arf MIS38 A10(ILE)
Nep H1h A10(ILE)
Oak FW305-7 A10(ILE)
Ban B1M1-1 A8(ILE/VAL)
Ban COW3 A8(ILE)
Ban BW11P2 A8(ILE)
Ban B1M1-15 A8(ILE)
Mdn LMG27940 A8(ILE)
Mdn SWRI103 A8(ILE)
Pek LMG26867 A8(ILE)
Pso WCS358 A11(ILE/VAL/LEU)
Pso PCL1445 A11(ILE/VAL/LEU)
Pso LMG11722 A11(ILE/VAL/LEU)
Pso WCU_64 A11(ILE/VAL/LEU)
Pso COR19 A11(ILE/VAL/LEU)
ThfB DSM11579 A5(VAL)
ThfB DSM11579 A8(THR)
Syf DC3000 A5(THR)
Tol NCPPB2192 A17(DAB)
Ses CMR12a A17(DAB)
Taa DSM16734 A17(DAB)
Xtl COR22 A12(LEU)
Xtl BW11M1 A12(LEU)
Xtl COR51 A12(LEU)
Xtl LMG27941 A12(LEU)
Xtl COW39 A12(LEU)
Xtl RW9S1A A12(LEU)
Etl L48 A12(LEU)
Cif SF1-54 A7(LEU)
Syf DC3000 A7(LEU)
Vif QS1027 A7(LEU)
Ppz Wu6 A8(LEU)
Ppz CR32 A8(LEU)
Poa RE*1-1-14 A8(LEU)
Coc B1M2-19 A9(LEU)
Gam Pf0-1 A9(LEU)
Ofa Pf-5 A8(LEU)
Ofa CHA0 A8(LEU)
Ofa PH1b A8(LEU)
Ofa CMR5c A8(LEU)
Ofa CMR12a A8(LEU)
B
Ten ZA5.3 C10
Mlk UCMA17988 C10
Ams DSS73 C10
Ste FhG100052 C10
Ten OE48.2 C10
Ani HKI0770 C10
Oak FW305-7 C10
Arf MIS38 C10
Lok COR10 C10
Nep H1h C10
Ams DSS73 C11
Ani HKI0770 C11
Arf MIS38 C11
Lok COR10 C11
Nep H1h C11
Oak FW305-7 C11
Mlk UCMA17988 C11
Ten ZA5.3 C11
Ten OE48.2 C11
Ste FhG100052 C11
Mlk UCMA17988 C8
Ten ZA5.3 C8
Ten OE48.2 C8
Ste FhG100052 C8
Ams DSS73 C8
Oak FW305-7 C8
Arf MIS38 C8
Lok COR10 C8
Ani HKI0770 C8
Nep H1h C8
Coc B1M2-19 C9
Gam Pf0-1 C9
Coc B1M2-19 C10
Gam Pf0-1 C10
Ban COW3 C8
Ban B1M1-1 C8
Ban BW11P2 C8
Ban B1M1-15 C8
Mdn SWRI103 C8
Mdn LMG27940 C8
Vsm DR54 C8
Vsm A2W4.9 C8
Mass SS101 C8
Pmn P867 C8
Pmn BRG-100 C8
Ppz Wu6 C9
Ppz CR32 C9
Poa RE*1-1-14 C9
Wip PGSB7828 C8
ViscC SBW25 C8
Wip LMG19695 C8
WipC LMG5329 C8
Ppz Wu6 C8
Ppz CR32 C8
Poa RE*1-1-14 C8
Vsa LMG22119 C8
Vsa LMG23076 C8
Vis OE28.3 C8
Vsa LMG 22709 C8
Vis LMG1794 C8
Pse NCPPB2192 C8
Vis SH10-3B C8
Wly LMG27247 C8
Viy BBc6R8 C8
Xtl COR22 C12
Xtl COR51 C12
Xtl BW11M1 C12
Xtl LMG27941 C12
Xtl RW9S1A C12
Xtl COW39 C12
Etl L48 C12
Pso PCL1445 C11
Pso WCS358 C11
Pso WCU_64 C11
Pso COR19 C11
Pso LMG11722 C11
Wlf COR54 C8
Wlf COW40 C8
Cif SF1-54 C7
Syf DC3000 C7
Vif QS1027 C7
Xtl COR22 C13
Xtl COR51 C13
Xtl BW11M1 C13
Xtl LMG27941 C13
Xtl COW39 C13
Xtl RW9S1A C13
Etl L48 C13
Pso PCL1445 C12
Pso WCS358 C12
Pso LMG11722 C12
Pso COR19 C12
Pso WCU_64 C12
Wlp RW1052 C8
Wlp NSE1 C8
Wpp JBCS1880 C8
Syf DC3000 C5
Cif SF1-54 C5
Wlc PB-St2 C8
Wlc LMG21630 C8
Pdm COR52 C8
Pek LMG26867 C8
Ofa CMR5c C8
Ofa CMR12a C8
Ofa PH1b C8
Ofa Pf-5 C8
Ofa CHA0 C8
Ofa CHA0 C9
Ofa Pf-5 C9
Ofa PH1b C9
Ofa CMR5c C9
Ofa CMR12a C9
Ses CMR12a C16
Tol NCPPB2192 C16
Taa DSM16734 C16
Tol NCPPB2192 C17
Ses CMR12a C17
Taa DSM16734 C17
Vif QS1027 C5
Thf DSM11579 C8
Thf DSM11579 C5
Thf DSM11579 C7
